# Extracting replicable associations across multiple studies: Empirical Bayes algorithms for controlling the false discovery rate

**DOI:** 10.1371/journal.pcbi.1005700

**Published:** 2017-08-18

**Authors:** David Amar, Ron Shamir, Daniel Yekutieli

**Affiliations:** 1 The Blavatnik School of Computer Science, Tel Aviv University, Tel Aviv, Israel; 2 Department of Statistics and OR, Tel Aviv University, Tel Aviv, Israel; Cornell University, UNITED STATES

## Abstract

In almost every field in genomics, large-scale biomedical datasets are used to report associations. Extracting associations that recur across multiple studies while controlling the false discovery rate is a fundamental challenge. Here, we propose a new method to allow joint analysis of multiple studies. Given a set of p-values obtained from each study, the goal is to identify associations that recur in at least *k* > 1 studies while controlling the false discovery rate. We propose several new algorithms that differ in how the study dependencies are modeled, and compare them and extant methods under various simulated scenarios. The top algorithm, SCREEN (Scalable Cluster-based REplicability ENhancement), is our new algorithm that works in three stages: (1) clustering an estimated correlation network of the studies, (2) learning replicability (e.g., of genes) within clusters, and (3) merging the results across the clusters. When we applied SCREEN to two real datasets it greatly outperformed the results obtained via standard meta-analysis. First, on a collection of 29 case-control gene expression cancer studies, we detected a large set of consistently up-regulated genes related to proliferation and cell cycle regulation. These genes are both consistently up-regulated across many cancer studies, and are well connected in known gene networks. Second, on a recent pan-cancer study that examined the expression profiles of patients with and without mutations in the HLA complex, we detected a large active module of up-regulated genes that are both related to immune responses and are well connected in known gene networks. This module covers thrice more genes as compared to the original study at a similar false discovery rate, demonstrating the high power of SCREEN. An implementation of SCREEN is available in the supplement.

## Author summary

When analyzing results from multiple studies, extracting replicated associations is the first step towards making new discoveries. The standard approach for this task is to use meta-analysis methods, which usually make an underlying null hypothesis that a gene has no effect in all studies. On the other hand, in replicability analysis we explicitly require that the gene will manifest a recurring pattern of effects. In this study we develop new algorithms for replicability analysis that are both scalable (i.e., can handle many studies) and allow controlling the false discovery rate. We show that our main algorithm called SCREEN (Scalable Cluster-based REplicability ENhancement) outperforms the other methods in simulated scenarios. Moreover, when applied to real datasets, SCREEN greatly extended the results of the meta-analysis, and can even facilitate detection of new biological results.

## Introduction

Confidence in reported findings is a prerequisite for advancing any scientific field. Such confidence is achieved by showing replication of discoveries in new studies [1]. In recent years studies have shown low reproducibility of results in several domains, including economics [2], psychology [3], medicine [4], and biology [5–7]. A new methodology called replicability analysis was recently suggested as a way to statistically pinpoint replicated discoveries across studies while controlling for the false discovery rate (FDR) [8]. This type of analysis is essential when trying to detect new hypotheses by integration of existing data from multiple high-throughput experiments.

The practical importance of replicability analysis is twofold. First, it quantifies the reliability of reported results. Second, collated information from multiple studies can identify scientific results that are beyond the reach of each single study. Indeed, in Genome Wide Association Studies (GWAS) replicability analysis allowed detection of new results that were not identified in meta-analysis, demonstrating that the two approaches are complementary [9].

Meta-analyses are widely applied and have been extensively studied in statistics [10] and in computational biology [11, 12]. However, in recent years the changes in the scale and scope of public high-throughput biomedical data have posed new methodological challenges. The first, and more obvious, is accounting for inflation in the number of false discoveries due to the multiplicity of outcomes, as hundreds of thousands and even millions of hypotheses are tested (see Zeggini et al. [13] for example). The second challenge is directly assessing consistency of results, which is not addressed by the classic null hypothesis of meta-analysis that the effect size is 0 in all the studies. Third, there is a need to distinguish between true effects that are specific to a single study and true effects that represent general discoveries and thus are replicable. For example, Kraft et al. [14] suggested that the effect of common genetic variants on the phenotype may correlate with population biases in a specific GWAS. While these are real discoveries in the sense that similar estimated effects are expected to be observed if the experiment could be replicated on the same cohort, their scientific importance is limited because they are specific to that cohort. For this reason, the authors argue that it is important to identify the association in additional studies conducted using a similar, but not identical, study base.

In recent years several frequentist approaches were suggested for the problem. Benjamini and Heller [15] introduced an inferential framework for replicability that is based on tests of partial conjunction null hypotheses. For meta-analysis of *n* studies of the same *m* outcomes and *u* = 1…*n*, the partial conjunction *H*^*u*/*n*^(*g*) is that outcome *g* has a non-null effect in less than *u* studies. Thus *H*^1/*n*^(*g*) is the standard meta-analysis null hypothesis that outcome *g* has a null effect in all *n* studies. The authors introduced p-values for testing *H*^*u*/*n*^(*g*) for each outcome. Benjamini, Heller and Yekutieli [8] applied the Benjamini-Hochberg FDR procedure [16] (BH) to the partial conjunction hypotheses p-values, and suggested setting *u* = 2 in order to assess replicability. Heller et al. [17] developed an approach for checking if a follow-up study corroborates the results reported in the original study. Song and Tseng [18] proposed a method to evaluate the proportion of non-null effects of a gene. However, they used the standard meta-analysis null hypothesis and their method cannot handle composite hypotheses, which are partial conjunctions *H*^*u*/*n*^ with *u* > 1.

Bayesian methods handle these shortcomings and offer a powerful framework for replicability analysis. For analyzing results from a single study, Efron introduced an empirical Bayes framework called the two-groups model [19]. It allows explicit analysis of the distribution of the statistic (e.g., p-values) of the underlying null and non-null groups. This clustering-based structure is then used to quantify the FDR of a rejection rule, and to compute a single point statistic, which is referred to as local Bayes FDR, or simply *fdr*. Heller and Yekutieli [9] introduced a method called repfdr, which extends the two-groups model for testing the partial conjunction hypotheses to the multi-study case. Formally, the problem is as follows: given an *n* × *m* matrix *Z*, where *Z*_*i*,*j*_ is the p-value (or z-score) of object (e.g., gene) *i* in study *j*, our goal is to identify the objects that are k-replicable (i.e., significant in *k* or more studies) while controlling the fdr.

Repfdr estimates the posterior probabilities of the various configurations of outcome effect status (null or non-null) across studies, and computes the fdr for each partial conjunction null by summing the posterior probabilities for the relevant configurations. The authors showed that their approach controls the FDR and offers more power than the frequentist methods. However, repfdr is not scalable and can only handle a few datasets. In addition, it was particularly designed for GWAS datasets in which the number of tested objects (e.g., SNPs) is very large (i.e., > 100k).

In this study, we propose ways to overcome the limitations of repfdr. Our focus is on allowing efficient computation when *m* is large and *n* is limited, such as in gene expression datasets (i.e., *n* ∼ 20k). To reach this goal we make three simplifying assumptions: (1) we ignore the effect size, (2) we ignore the direction of the statistic, and (3) we assume that the studies originate from independent clusters. In addition, to handle larger values of *m* we compute an upper bound for the *fdr*, cutting the running time substantially. Our main algorithm is called SCREEN (Scalable Cluster-based REplicability ENhancement). It first detects the study clusters, then uses Expectation-Maximization (EM) to model each cluster, and finally merges the clusters using dynamic programming. Other algorithms that we propose here include two variants of SCREEN that differ in the way the studies are clustered: *SCREEN*–*ind* assumes independence and treats each study as a single cluster, and repfdr-UB puts all studies in one cluster. We compared SCREEN to other algorithms using various simulated scenarios and showed that only SCREEN had consistently low empirical false discovery proportions, and very high detection power.

We applied SCREEN to two cancer datasets, where each is a collection of case-control gene expression experiments. In both cases SCREEN greatly improved the results obtained by standard meta-analysis, and provided new biological insights. The first dataset is a collection of 29 case-control gene expression cancer studies from different tissues. Here, SCREEN detected a large set of genes that are consistently up-regulated, highly enriched for cell proliferation and cell cycle regulation functions, and are well connected in known gene networks, indicating their functional coherence. The second dataset is a recent pan-cancer study that examined the expression profiles of patients with and without mutations in the HLA complex across 11 cancer types [20]. SCREEN detected a large set of up-regulated genes that are related to immune responses. Importantly, SCREEN reported many more immune response genes than the original study thanks to our ability to quantify the fdr, and allowed detection of prominent genes and pathways that were not reported previously.

## Results

Outline: After introducing some background and notation, we present a dynamic programming algorithm for calculating the fdr under the assumption that the studies are independent. Second, we discuss EM-based algorithms for dealing with dependence. We then show that given the prior probability of only a subset of all configurations an upper-bound for the *fdr* can be computed. This leads us to a much faster algorithm that can handle many studies. Third, we extend the dynamic programming algorithm to handle independent clusters of studies. This will lead us to the complete SCREEN algorithm, which is based on EM-based dependency modeling within study clusters and merging the results using dynamic programming. Finally, we discuss experimental results on simulated and real datasets.

### Preliminaries and notations

We start with a brief introduction to the single-study model. For a full description and background see [19]. Given a large set of *N* hypotheses tested in a large-scale study, the two-groups model provides a simple Bayesian framework for multiple testing: each of the *N* cases (e.g., genes in a gene expression study) are either null or non-null with prior probability *π*_0_ and *π*_1_ = 1 − *π*_0_, and with z-scores (or p-values) having density either *f*_0_(*z*) or *f*_1_(*z*). When the assumptions of the statistical test are valid, we know that the *f*_0_ distribution is a standard normal (or a uniform distribution for p-values), and we call it the theoretical null. The mixture density and probability distributions are:

$$\begin{array}{c} f\left(z\right)=\pi_{0}f_{0}\left(z\right)+\pi_{1}f_{1}\left(z\right)\\ F\left(z\right)=\pi_{0}F_{0}\left(z\right)+\pi_{1}F_{1}\left(z\right) \end{array}$$

For a rejection area ${Ƶ}_{y}=\left(-\infty ,y\right)$, using Bayes rule we get:

$$\begin{array}{c} Fdr\left({Ƶ}_{y}\right)\equiv Pr\left\{null|z\in {Ƶ}_{y}\right\}=\pi_{0}F_{0}\left(y\right)/F\left(y\right) \end{array}$$

We call *Fdr* the (Bayes) false discovery rate for Ƶ: this is the probability we would make a false discovery if we report Ƶ as non-null. If Ƶ is a single point *z*_0_ we define the local (Bayes) false discovery rate as:

$$\begin{array}{c} fdr\left(z_{0}\right)\equiv Pr\left\{null|z=z_{0}\right\}=\pi_{0}f_{0}\left(z_{0}\right)/f\left(z_{0}\right) \end{array}$$

Previous work have shown that: (1) the Bayes Fdr is tightly related to false discovery control in the frequentist sense, and (2) using a threshold on the local fdr for defining discoveries is equivalent to the optimal Bayes rule in terms of classification between nulls and non-nulls. Moreover, a threshold of 0.2 on the local fdr was suggested [19, 21]. Note that in our Bayesian setting computing the local fdr of a gene is an estimation problem. Within the context of our study we incorporated and tested two established methods for two-groups estimation studies: locfdr [22] and estimation based on mixture of Gaussians, which we call normix, see Materials and methods for details.

Consider an extension of the two group model to analysis of *n* genes over *m* > 1 studies. The data for gene *i* are a vector of m statistics *Z*_*i*,⋅_ = (*Z*_*i*,1_, ⋯, *Z*_*i*,*m*_) that are all either z-scores or p-values. For simplicity, from now on we assume that these data are z-scores. The unknown parameter for gene *i* = 1, ⋯, *n* is a binary configuration vector *H*_*i*,⋅_ = (*H*_*i*,1_, ⋯, *H*_*i*,*m*_), with *H*_*i*,*j*_ ∈ {0, 1}. If *H*_*i*,*j*_ = 0 then gene *i* is a *null* realization in study *j*, and it is a *non*–*null* realization otherwise.

We assume that in each study *j* the parameters of the two-groups model $\theta_{j}:(\pi_{0}^{j},f_{0}^{j},f_{1}^{j},f^{j})$ are fixed and focus on replicability analysis. Generally, unless mentioned otherwise, we assume that the genes are independent. However, note that estimation of *θ*_*j*_ can account for gene dependence within study *j* [23, 24]. Finally, we also assume that the z-scores of a gene are independent given its configuration. That is,

$$\begin{array}{c} P(Z_{i,\cdot }|H_{i,\cdot })=\prod_{j=1}^{m}P(Z_{i,j}|H_{i,j})=\prod_{j=1}^{m}{\left(f_{0}^{j}\left(Z_{i,j}\right)\right)}^{\left(1-H_{i,j}\right)}{\left(f_{1}^{j}\left(Z_{i,j}\right)\right)}^{H_{i,j}} \end{array}$$

Next, we use *h* ∈ {0, 1}^*m*^ to denote an arbitrary configuration vector, and *π*(*h*) to denote a probability assigned to the parameter space. We assume that the researcher has a set of configurations $H_{1}\subseteq {\left\{0,1\right\}}^{m}$ that represents the desired rejected genes. Here we will assume that $H_{1}$ corresponds to genes that are non-null in at least *k* studies: $H_{1}=\left\{h:|h|\geq k\right\}$, where $\left|h\right|=\sum_{j=1}^{m}h_{j}$.

As a note, selection of *k* depends on the research question at hand. For example, Heller and Yekutieli used *k* = 2 to detect minimal replicability of SNPs in a GWAS [9]. Low *k* values can also be reasonable if the *m* studies represent different biological questions that are related, such as differential expression experiments from different cancer subtypes. On the other hand, if the *m* studies represent tightly related experiments such as biological replicates then larger k (e.g., *m*/2) seems more reasonable.

The local false discovery rate (fdr) of a gene *i* can be formulated as:

$$\begin{array}{c} fdr\left(Z_{i,\cdot }\right)=Pr\left(\bar{H_{1}}|Z_{i,\cdot }\right)=\sum_{h:h\notin H_{1}}P\left(h|Z_{i,\cdot }\right)=\sum_{h:h\notin H_{1}}\frac{P\left(Z_{i,\cdot }|h\right)P\left(h\right)}{P(Z_{i,\cdot })} \end{array}$$

For a given *k* and $H_{1}=\left\{h:|h|\geq k\right\}$ we get:

$$\begin{array}{c} fdr_{k}\left(Z_{i,\cdot }\right)=\sum_{h:|h|<k}\frac{P\left(Z_{i,\cdot }|h\right)P\left(h\right)}{P(Z_{i,\cdot })} \end{array}$$

### An *O*(*mnk*) algorithm when studies are independent

We first address the case where studies are independent.

**Lemma.** If the studies are independent (in the parameter space) then:

$$\begin{array}{c} fdr_{k}\left(Z_{i,\cdot }\right)=\sum_{h:|h|<k}\prod_{j=1}^{m}\frac{P\left(Z_{i,j}|h_{j}\right)P\left(h_{j}\right)}{f^{j}\left(Z_{i,j}\right)} \end{array}$$

*Proof.* First, note that under the independence assumption $P\left(h\right)=\prod_{j=1}^{m}P\left(h_{j}\right)=\prod_{j=1}^{m}{\pi_{0}^{j}}^{\left(1-h_{j}\right)}{\left(1-\pi_{0}^{j}\right)}^{h_{j}}$. Second, as the z-scores are independent given the configuration vector *h* we get that:

$$\begin{array}{ccc} P(Z_{i,\cdot }) & = & \sum_{h}P\left(Z_{i,\cdot }|h\right)P\left(h\right)=\sum_{h}\prod_{j=1}^{m}P\left(Z_{i,j}|h_{j}\right){\pi_{0}^{j}}^{\left(1-h_{j}\right)}{\left(1-\pi_{0}^{j}\right)}^{h_{j}}\\ & = & \prod_{j=1}^{m}(P\left(Z_{i,j}|h_{j}=0\right)\pi_{0}^{j}+P\left(Z_{i,j}|h_{j}=1\right)\left(1-\pi_{0}^{j}\right)) \end{array}$$

**Proposition:** If the studies are independent then *fdr*_*k*_ can be computed in *O*(*mnk*).

*Proof*. By the lemma, the *fdr* of a gene is based on the product of the two-group model densities in each study. Therefore:

$$\begin{array}{c} fdr_{k}^{indep}\left(Z_{i,\cdot }\right)=\sum_{h:|h|<k}\;\prod_{j=1}^{m}\frac{{\left(\pi_{0}^{j}f_{0}^{j}\left(Z_{i,j}\right)\right)}^{1-h_{j}}{\left(\left(1-\pi_{0}^{j}\right)f_{1}^{j}\left(Z_{i,j}\right)\right)}^{h_{j}}}{f^{j}\left(Z_{i,j}\right)} \end{array}$$

We use dynamic programming to calculate *fdr*_*k*_(*z*_*i*_) as follows. Define:

$$\begin{array}{c} U\left[i,j,k^{*}\right]=\sum_{h:|h|=(k^{*}-1)}\;\prod_{j=1}^{m}\frac{{\left(\pi_{0}^{j}f_{0}^{j}\left(Z_{i,j}\right)\right)}^{1-h_{j}}{\left(\left(1-\pi_{0}^{j}\right)f_{1}^{j}\left(Z_{i,j}\right)\right)}^{h_{j}}}{f^{j}\left(Z_{i,j}\right)} \end{array}$$

These values can be calculated (for each gene *i*) by updating a table of *m* × (*k* + 1) values. The base cases are:

$$\begin{array}{c} U\left[i,j,1\right]=\prod_{j=1}^{m}\frac{\pi_{0}^{j}f_{0}^{j}\left(Z_{i,j}\right)}{f^{j}\left(Z_{i,j}\right)} \end{array}$$

The recursive formulas are:

$$\begin{array}{ccc} U[i,j,k^{*}] & = & \frac{\pi_{0}^{j}f_{0}^{j}\left(Z_{i,j}\right)}{f^{j}\left(Z_{i,j}\right)}U\left[i,j-1,k^{*}\right]+\frac{{\left(1-\pi_{0}\right)}^{j}f_{1}^{j}\left(Z_{i,j}\right)}{f^{j}\left(Z_{i,j}\right)}U\left[i,j-1,k^{*}-1\right] \end{array}$$

Finally, to obtain the *fdr* of a gene we sum over the values in the last column:

$$\begin{array}{c} fdr_{k}^{indep}\left(Z_{i,\cdot }\right)=\sum_{k^{*}=1}^{k-1}U\left[i,m,k^{*}\right] \end{array}$$

The running time for analyzing each gene is *O*(*mk*) and the total running time is *O*(*nmk*).

### Schemes for handling dependencies

The empirical Bayes method of [9] estimates the prior distribution *π*(*h*) directly from the data. This approach has two drawbacks. First, the EM algorithm explicitly keeps a value for each possible configuration, which makes the algorithm intractable when *m* is large. Second, the estimation for rare configurations might be inaccurate, unless *n* >>2^*m*^. As an alternative, we develop an algorithm that keeps in memory only a small set of high probability configurations. We then use these estimates to obtain an upper bound for the *fdr* of a gene.

### The unrestricted EM algorithm

We first describe the EM in the full configuration space, and then modify it for the constrained case. That is, the EM is guaranteed to improve the solution and converge. The unrestricted EM formulation is based on repfdr [9, 25], as follows:

The E-step:

$$\begin{array}{c} P\left(H_{i,\cdot }=h|Z_{i,\cdot },\pi^{\left(t\right)}\left(h\right)\right)=\frac{f\left(Z_{i,\cdot }|h\right)\pi^{\left(t\right)}\left(h\right)}{\sum_{h^{\prime }}f\left(Z_{i,\cdot }|h^{\prime }\right)\pi^{\left(t\right)}\left(h^{\prime }\right)} \end{array}$$

The M-step:

$$\begin{array}{c} \pi^{\left(t+1\right)}\left(h\right)=\frac{1}{n}\sum_{i}P\left(H_{i,\cdot }=h|Z_{i,\cdot },\pi^{\left(t\right)}\left(h\right)\right)=\frac{1}{n}\sum_{i}\frac{f\left(Z_{i,\cdot }|h\right)\pi^{\left(t\right)}\left(h\right)}{\sum_{h^{\prime }}f\left(Z_{i,\cdot }|h^{\prime }\right)\pi^{\left(t\right)}\left(h^{\prime }\right)} \end{array}$$

This process guarantees convergence to a local optimum. Our goal is to limit the search space.

**Lemma.** The EM algorithm above can be used to find a local optimum estimator under the constraint $\forall h\notin H^{\prime }\;\pi \left(h\right)=0$, for any non-empty configuration set $H^{\prime }$.

*Proof*. Note that during the EM iterations, if at some time point *t* *π*^(*t*)^(*h*) = 0 then ∀t* > *t* *π*^(t*)^(*h*) = 0. Therefore, setting the starting point of the EM such that $\forall h\notin H^{\prime }\;\pi^{\left(0\right)}\left(h\right)=0$ satisfies the constraint and ensures convergence.

### The restricted EM

In this section we present a restricted version of the EM above, which we call repfdr-UB. To describe it, we need additional notation. Given a configuration vector *h* ∈ {0, 1}^*m*^, let *h*[*l*] be the vector containing the first *l* entries of *h*. Given a real valued vector *v*, let *v*_(*i*)_ denote the i’th smallest element of *v*.

Our algorithm is based on the simple observation that if *π*(*h*[*l*]) ≤ *ϵ* then any extension of *h*[*l*] cannot exceed *ϵ*. That is, *π*(*h*[*l* + 1]) ≤ *ϵ* regardless of the new value in study *l* + 1. Our algorithm works as follows. The user specifies a limit to the number configurations kept in the memory—*n*_*H*_. For simplicity we assume that *n*_*H*_ is a power of 2. We first run the unrestricted EM algorithm on the first *log*_2_(*n*_*H*_) − 1 studies. Each subsequent iteration adds a new study. In each iteration *l* we keep four parameters: (1) ${\hat{H}}^{l}$—the set of the top *n*_*H*_ probability configurations, (2) ${\hat{\pi }}^{l}$—the vector of their assigned probabilities, (3) ${\hat{\xi }}^{l}$—an estimation of $\sum_{h\left[l\right]\in {\hat{H}}^{l}}\pi^{l}\left(h\left[l\right]\right)$, and (4) ${\hat{\epsilon }}^{l}$—an estimation of the maximal probability among the excluded configurations.

Initially, *l* = *log*_2_(*n*_*H*_) − 1 and ${\hat{H}}^{l}$ contains all possible configurations of the first *l* studies. In addition ${\hat{\xi }}^{l}=1$, and ${\hat{\epsilon }}^{l}=0$. In iteration *l* + 1 we run the restricted EM algorithm on all possible extensions of ${\hat{H}}^{l}$. That is, the input configuration set for the EM is a result of adding either 1 or 0 at the *l* + 1 position of each configuration in ${\hat{H}}^{l}$. The EM run produces initial estimations for our parameters, on which the following ordered updates are applied:

$$\begin{array}{ccc} {\hat{\pi }}^{l+1} & = & {\hat{\xi }}^{l}{\hat{\pi }}^{l+1} \end{array}$$ (1)

$$\begin{array}{ccc} {\hat{H}}^{l+1} & = & \left\{h\left[l+1\right];{\hat{\pi }}^{l+1}\left(h\left[l+1\right]\right)\geq {\hat{\pi }}_{\left(n_{H}/2\right)}^{l+1}\right\} \end{array}$$ (2)

$$\begin{array}{ccc} {\hat{\xi }}^{l+1} & = & \sum_{h\left[l+1\right]\in {\hat{H}}^{l+1}}\pi^{l+1}\left(h\left[l+1\right]\right) \end{array}$$ (3)

$$\begin{array}{ccc} {\hat{\epsilon }}^{l+1} & = & \max({\hat{\epsilon }}^{l},\max_{h\left[l+1\right]\notin {\hat{H}}^{l+1}}\left({\hat{\pi }}^{l+1}\left(h\left[l+1\right]\right)\right)) \end{array}$$ (4)

Note that in step Eq (2) above we keep the top *n*_*H*_/2 configurations in ${\hat{H}}^{l+1}$. This set is then used as input to the EM run in the next iteration. We repeat the process above until *l* = *m*. The output of the algorithm is ${\hat{H}}^{m},{\hat{\pi }}^{m},{\hat{\xi }}^{m},{\hat{\epsilon }}^{m}$.

#### A fast algorithm for an upper bound for the fdr

We now show that the restricted algorithm provides an upper bound for the *fdr*_*k*_ of a gene in running time *O*(*m*(*k* + *n*_*H*_)) for each gene.

**Theorem.** Given the prior probability *π*(*h*) of each configuration *h* in $H^{\prime }\subseteq H$, and an upper bound *ϵ* for the probability of all excluded configurations, the following inequality holds:

$$\begin{array}{c} fdr_{k}\left(Z_{i,\cdot }\right)\leq \frac{\sum_{h:|h|<k\wedge h\in H^{\prime }}P\left(Z_{i,\cdot }|h\right)\left(\pi \left(h\right)-\epsilon \right)+\epsilon \sum_{h:|h|<k}P\left(Z_{i,\cdot }|h\right)}{\sum_{h\in H^{\prime }}P\left(Z_{i,\cdot }|h\right)\pi \left(h\right)} \end{array}$$

*Proof*. Given that for each $h\notin H^{\prime }\;\pi (h)\leq \epsilon$, we get:

$$\begin{array}{ll} fdr_{k}\left(Z_{i,\cdot }\right)= & \sum_{h:\left|h\right|<k\wedge h\in H^{\prime }}P(h|Z_{i,\cdot })+\sum_{h:\left|h\right|<k\wedge h\notin H^{\prime }}P(h|Z_{i,\cdot })\leq \\ & \begin{array}{c} \sum_{h:\left|h\right|<k\wedge h\in H^{\prime }}P(h|Z_{i,\cdot })+\epsilon \left(\sum_{h:\left|h\right|<k\wedge h\notin H^{\prime }}\frac{P(Z_{i,\cdot }|h)}{P\left(Z_{i,\cdot }\right)}\right) \end{array} \end{array}$$

Thus:

$$\begin{array}{ll} fdr_{k}\left(Z_{i,\cdot }\right)\leq & \sum_{h:\left|h\right|<k\wedge h\in H^{\prime }}\frac{P(Z_{i,\cdot }|h)\pi \left(h\right)}{P\left(Z_{i,\cdot }\right)}+\epsilon \left(\sum_{h:\left|h\right|<k\wedge H\notin H^{\prime }}\frac{P(Z_{i,\cdot }|h)}{P\left(Z_{i,\cdot }\right)}\right)=\\ & \sum_{h:\left|h\right|<k\wedge h\in H^{\prime }}\frac{P(Z_{i,\cdot }|h)\left(\pi \left(h\right)-\epsilon \right)}{P\left(Z_{i,\cdot }\right)}+\epsilon \left(\sum_{h:\left|h\right|<k}\frac{P\left(Z_{i,\cdot }h\right)}{P\left(Z_{i,\cdot }\right)}\right) \end{array}$$

Finally, since $P\left(Z_{i,\cdot }\right)=\sum_{h}P\left(Z_{i,\cdot }|h\right)\pi \left(h\right)\geq \sum_{h\in H^{\prime }}P\left(Z_{i,\cdot }|h\right)\pi \left(h\right)$, we obtain:

$$\begin{array}{c} fdr_{k}\left(Z_{i,\cdot }\right)\leq \frac{\sum_{h:|h|<k\wedge h\in H^{\prime }}P\left(Z_{i,\cdot }|h\right)\left(\pi \left(h\right)-\epsilon \right)+\epsilon \sum_{h:|h|<k}P\left(Z_{i,\cdot }|h\right)}{\sum_{h\in H^{\prime }}P\left(Z_{i,\cdot }|h\right)\pi \left(h\right)} \end{array}$$

**Corollary.** The restricted algorithm provides an upper bound for the *fdr*_*k*_ of a gene in running time *O*(*m*(*k* + *n*_*H*_)) for each gene.

*Proof*. The final term in the proof above can be calculated in *O*(*m*(*n*_*H*_ + *k*)). First, the terms $\sum_{h:|h|<k\wedge h\in H^{\prime }}P\left(Z_{i,\cdot }|h\right)\left(\pi \left(h\right)-\epsilon \right)$ and $\sum_{h\in H^{\prime }}P\left(Z_{i,\cdot }|h\right)\pi \left(h\right)$ are calculated directly using the output of our EM-like algorithm in *O*(*mn*_*H*_). Then, *ϵ*∑_*h*:|*h*|<*k*_ *P*(*Z*_*i*,⋅_|*h*) can be calculated using dynamic programming in a similar fashion to our algorithm for calculating *fdr*_*k*_ under independence assumption. Thus, the total running time for all genes, given the output of the EM, is *O*(*mn*(*n*_*H*_ + *k*)).

### Replicability across independent study clusters

In this section we apply the ideas from the previous sections to obtain an algorithm for calculating the *fdr* under the assumption that the studies originate from independent clusters. We call this algorithm SCREEN (Scalable Cluster-based REplicability ENhancement, see S1 Text for an overview of the method). Briefly, our algorithm has three stages. First, we use the EM-process on each study pair to create a network of study correlations. We then cluster the network to obtain a set of study clusters that are likely to be independent, see Materials and methods for the full description of this step. Second, we run the EM on each cluster separately. Finally, we merge the results from the different clusters using dynamic programming. Note that this algorithm is a heuristic as it uses EM within each cluster.

#### A fast algorithm for combining study clusters

Assume for now that we are given a clustering of the studies into *M* independent clusters *C*_1_, ⋯, *C*_*M*_. Thus:

$$\begin{array}{c} \pi \left(h\right)=\prod_{j=1}^{M}P\left(h_{C_{j}}\right) \end{array}$$

where $h_{C_{j}}$ denotes the subvector of *h* confined to the studies in the cluster *C*_*j*_. Let $Z_{i,C_{j}}$ be the z-scores of gene *i* in the studies of cluster *C*_*j*_, then *fdr*_*k*_ has the following form:

$$\begin{array}{c} \sum_{h:|h|<k}\;\prod_{j=1}^{M}\frac{P\left(Z_{i,C_{j}}|h_{C_{j}}\right)P\left(h_{C_{j}}\right)}{P(Z_{i,C_{j}})} \end{array}$$

This form is a generalization of the formulation under indepepndence assumption, which implies that the dynamic programming approach can be used to merge data across clusters. We now describe the full method.

We apply our EM approach to each cluster separately, and calculate the probability that gene *i* has exactly k* non-null realizations in cluster *C*_*j*_:

$$\begin{array}{c} V_{C_{j}}\left[i,k^{*}\right]=\sum_{h_{C_{j}}:\left|h_{C_{j}}\right|=k^{*}-1}P\left(h_{C_{j}}|Z_{i,C_{j}}\right) \end{array}$$

Let *V*[*i*, *j*, k*] be the probability that gene *i* has k* − 1 non-null realizations over clusters 1, ⋯, *j*. Then:

$$\begin{array}{lll} V\left[i,1,k^{*}\right] & = & \{\begin{array}{cc} V_{C_{1}}\left[i,k^{*}\right] & \text{if }k^{*}<\left|C_{1}\right|\\ 0 & \text{otherwise} \end{array}\\ V\left[i,j,k^{*}\right] & = & \sum_{k^{\prime }=1}^{min\left(k^{*},\left|C_{j}\right|\right)}V_{C_{j}}\left[i,k^{\prime }\right]V\left[i,j-1,k^{*}-k^{\prime }\right]\\ fdr_{k}\left(Z_{i,\cdot }\right) & = & \sum_{k^{*}=1}^{k-1}V\left[i,m,k^{*}\right] \end{array}$$

The table *V* above has *M* × *k* entries for each gene *i*, and the update rule takes *O*(*k*). Thus, given the EM results in each cluster, the running time of this algorithm is *O*(*k*^2^*M*) for each gene.

### Experimental results

#### Simulations

Compared methods: We evaluated six different approaches for replicability. (1) Fisher: Fisher’s meta-analysis for each gene with BH correction. (2) BH-count: the number of q-values ≤ 0.1 for each gene after applying BH in each study. (3) Exp-count: an estimator for the expected number of non-nulls. See Materials and methods for details on 1-3; (4) Our algorithm for calculating *fdr*_*k*_ exactly under independence assumption, which we call SCREEN-ind, (5) repfdr-UB, which computes an upper bound for *fdr*_*k*_ in order to handle many possibly dependent studies, and (6) SCREEN, which evaluates replicability across study clusters. For 3-6, we tested two methods for two-groups estimation within studies: locfdr [22] and estimation based on mixture of Gaussians, which we call normix, see Materials and methods for details.

Simulation overview: We simulated scenarios of independent and dependent studies as explained later. In each of the scenarios below we started by creating a pair of matrices, *P*, and *H*^*P*^. The number of genes *n* was 5000 and the number of studies *m* varied (in all scenarios *m* ≥ 20). *P* is a matrix of p-values, and $H_{i,j}^{P}\in \left\{0,1\right\}$ denotes whether the p-value of gene *i* in study *j* is from the null group ($H_{i,j}^{P}=0$) or the non-null group ($H_{i,j}^{P}=1$). We first generated *H*^*P*^, and then determined *P* given the gene configurations in *H*^*P*^ as follows. For each *i*, *j* where $H_{i,j}^{P}=0$ the value *P*_*i*,*j*_ was randomly selected from a uniform distribution. For each cell *i*, *j* for which $H_{i,j}^{P}=1$ the p-value was drawn with probability 0.5 from *β*(1, *x*) (i.e., low p-values), and otherwise from *β*(*x*, 1) (i.e., high p-values). We tested *x* = 10, 100, and 1000, corresponding to distributions with 0.1, 0.01, and 0.001 mean p-values, respectively. In addition to the non-null distributions we also varied other parameters in the creation of *H*^*P*^: the number of non-nulls, and the correlation among the studies (i.e., the columns of *H*^*P*^).

Performance evaluation: We tested the ability of the methods to detect genes that are non-null in two or more studies. Here, the unknown parameter of a gene was the number *k* of 1 values in its row in *H*^*P*^. For each *k* between 2 and 5 we ran all algorithms above. For repfdr-UB we set the number of configurations *n*_*H*_ to 512. In methods that are based on calculating the local fdr we used a threshold of 0.2 for selecting the genes. For methods that are based on counting we used *k* as a threshold. For Fisher’s meta-analysis we used *q* ≤ 0.1 as a threshold. For every *k* we compared the genes identified by each algorithm to the set of genes with at least *k* non-null realizations (given in *H*^*P*^). In each of the cases below we calculated the empirical false discovery proportion (FDP; i.e., the proportion of erroneously declared non-nulls). For methods that had *FDP* < 0.2 across all tested *k* values we also calculated the Jaccard score in order to quantify the overall agreement between the output and the known solution. Thus, only methods that consistently performed well in terms of FDP are evaluated in their detection power.

#### Scenario 1: Independent studies

Here, a random set of 300 genes were selected to be non-nulls independently in each study. In addition, we selected 50 genes to have non-null *β*(1, *x*) p-values in 5 additional studies. These genes were added to ensure presence of a large set of replicable genes with high *k* values (such a set could arise e.g., from up regulation of a pathway).

**Fig 1:**
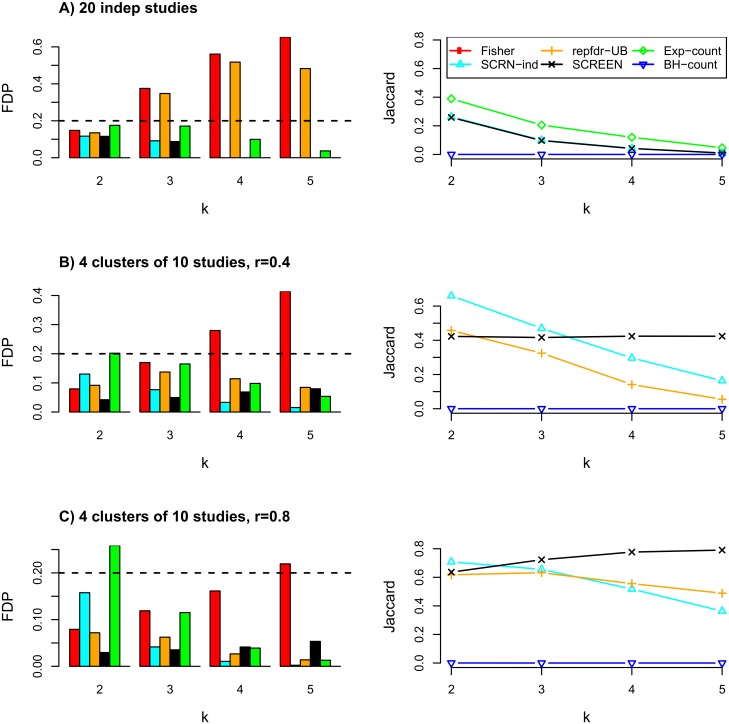
Simulation results: Independent and dependent studies. A) 20 independent studies. B) Four clusters of 10 studies each with a low correlation within each cluster. C) Four clusters of 10 studies each with a high correlation within each cluster. The non-null distribution in each case was Beta(1,100). Two-groups estimation was done using normix. The left column shows the empirical FDP (values above 0.4 are not shown). The right column shows the Jaccard scores only for methods that had consistently low FDP values (< 0.2) for all *k* values. BH-count, SCREEN and SCREEN-ind (SCRN-ind for short) are the only methods that had *FDP* ≤ 0.2 in all cases. BH-count had very low FDP but also very low Jaccard scores. Except for *k* = 2, SCREEN had similar or better performance compared to SCREEN-ind.

Fig 1A shows the results for *x* = 100 using normix. Fisher’s method and repfdr-UB had high FDP for *k* ≥ 3. Exp-count had better Jaccard than SCREEN and SCREEN-ind, but at the expense of a higher FDP (which reached almost 0.15). For all tested *x* values the results using locfdr and normix were very similar, and SCREEN was very similar to SCREEN-ind as it tended to correctly cluster each study separately (see Fig 1 and S1–S3 Figs). For *x* = 1000 (S1 Fig) SCREEN and SCREEN-ind had the best Jaccard for almost every *k* and *FDP* ≤ 0.1. In contrast, Fisher’s method had very high FDP (≥ 0.25), again illustrating why standard meta-analysis is not suitable for our goal. For *x* = 10 the *FDP* scores of repfdr-UB were high (e.g., ≥ 0.25) for each *k* as well as the FDP scores of *SCREEN* for *k* = 2 using the normix method. All other FDP scores were very close to zero, and when the FDP values of SCREEN were high, very few genes where reported(≤ 4), see S3 Fig.

#### Scenario 2: Dependent studies

We simulated data with *M* = 4 clusters of ten studies each. Here, the matrix *H*^*P*^ was generated by first creating an auxiliary matrix *A* of the same dimensions as *H*^*P*^. The rows of *A* were drawn independently from a multivariate normal distribution $N(0,\Sigma_{M})$, where *Σ*_*M*_ specified a correlation structure of *M* independent study clusters. Within each cluster the correlation was *r* = 0.8, or *r* = 0.4. *H*^*P*^ was created from *A* by setting a threshold such that the expected number of non-nulls in each study was 300. That is, $H_{i,j}^{P}=1$ if and only if *A*_*i*,*j*_ ≥ *τ*^*j*^, where *τ*^*j*^ is the 0.94-th quantile of the normal distribution of column *j* of *A*. Given *H*^*P*^, *P* was created as in Scenario 1 with *x* = 100.

**Fig 2:**
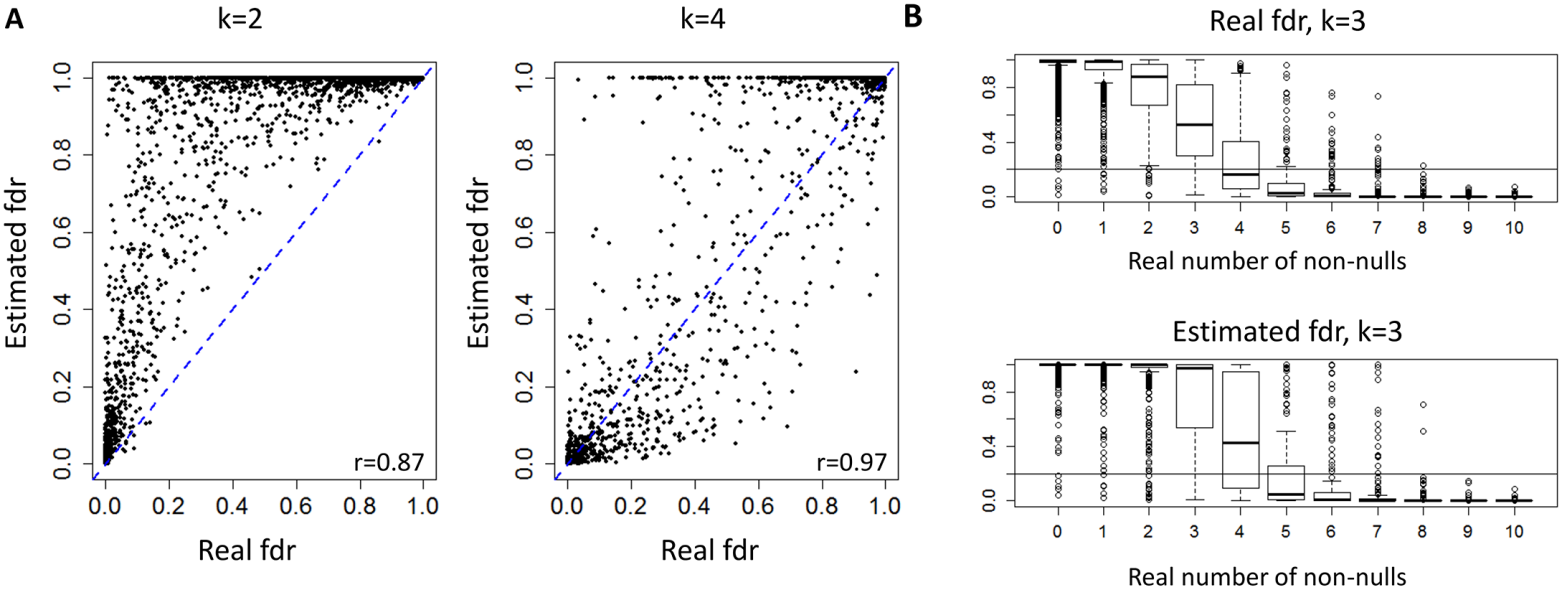
Simulation of dependent studies (4 clusters of 10 studies each). The figures show SCREEN’s estimations vs. real fdr values for different *k* values. The number of study clusters is 4 and the correlation within the clusters is set using *r* = 0.8. The non-null distribution within each study is *Beta*(1,100). A) Real vs. estimated fdr values for *k* = 2 and *k* = 4. Each dot corresponds for a gene. For *k* = 2 the estimated *fdr* values are higher, demonstrating stringent FDR control. For *k* = 4 the real and estimated values are highly correlated. B) fdr distributions as a function of the real number of non-nulls (y-axis is the fdr value). Top: real fdrs, bottom: estimated fdr. Each boxplot represents a different set of genes whose real number of non-nulls is given in the x-axis label (except for 10, which means at least 10 non-nulls).

The results using normix are summarized in Fig 1B and 1C. In terms of *FDP*, all algorithms except for Fisher’s method and Exp-count performed well. In terms of the Jaccard score SCREEN achieved top performance for *k* ≥ 4, and SCREEN-ind achieved top performance for *k* ≤ 3. As in the previous scenario, normix and locfdr gave similar results, see S2 Fig. Fig 2A shows the *fdr* values of SCREEN compared to the real fdr values for different *k* values (*r* = 0.8). For *k* = 4 show that our estimations are highly correlated with the real values. Fig 2B shows that the estimated fdr values decrease with the real number of non-nulls. In addition, most of SCREEN’s errors are made for genes with 3 or 4 non-null realizations, and the real fdr values will not necessarily capture these genes at *fdr*_*k*_ ≤ 0.2. On the other hand, a greater fdr threshold can be used (e.g., 0.4) to cover additional true negatives at the expense of a few false positives.

In summary, our simulations show that out of the *fdr*_*k*_-based methods, SCREEN-ind and SCREEN had consistently low FDP values, while achieving the highest Jaccard scores. SCREEN-ind had a slight advantage in low *k* values, whereas SCREEN was better in higher *k* values.

#### Scenario 3: Dense effects

In our simulations above the gene effects were sparse. That is, in all scenarios the non-null prior probability was relatively low. When we analyzed real datasets (see below) we observed that while some studies were in line with these classic assumptions, others were not, see S4 and S5 Figs. To cope with these cases of dense effects we performed extensive additional analysis on both simulated and real data, see S1 Text. Our main findings are as follows: (1) locfdr often fails to model these cases, (2) locfdr and normix with empirical null estimation overestimate the null prior probability, and (3) as reported in the previous section, using SCREEN with normix and a fixed theoretical null achieved very high Jaccard scores and low FDP on simulated data. We therefore use the latter approach to analyze the datasets in the subsequent sections.

### Analysis of cancer datasets

We analyzed two real datasets. The first, which we call Cancer DEG, is a collection of gene expression studies that compared cancer to non-cancer tissues. The second, called HLA, is from [20], where Shukla et al. tested differential expression between cancer samples with and without somatic mutations in the HLA complex across 11 TCGA cancer subtypes.

#### The cancer DEG dataset

This dataset contains 29 microarray gene expression studies that compared cancer to non-cancer tissues. It was selected from our previously published compendium [26] by taking all studies that had at least 10 cancer and 10 non-cancer samples (one study was excluded since its gene set was too small). For each dataset genes were assigned p-values for distinguishing between the cancer and non-cancer classes using the NCBI GEO2R web tool [27], where the p-values were calculated using a two-tailed t-test for differential expression. The resulting p-value matrix had 11540 rows (genes) and 29 columns. S6 Fig shows the estimated pairwise correlations between studies. SCREEN identified eight clusters: a single large cluster of 19 studies and 7 clusters with one or more studies.

**Fig 3:**
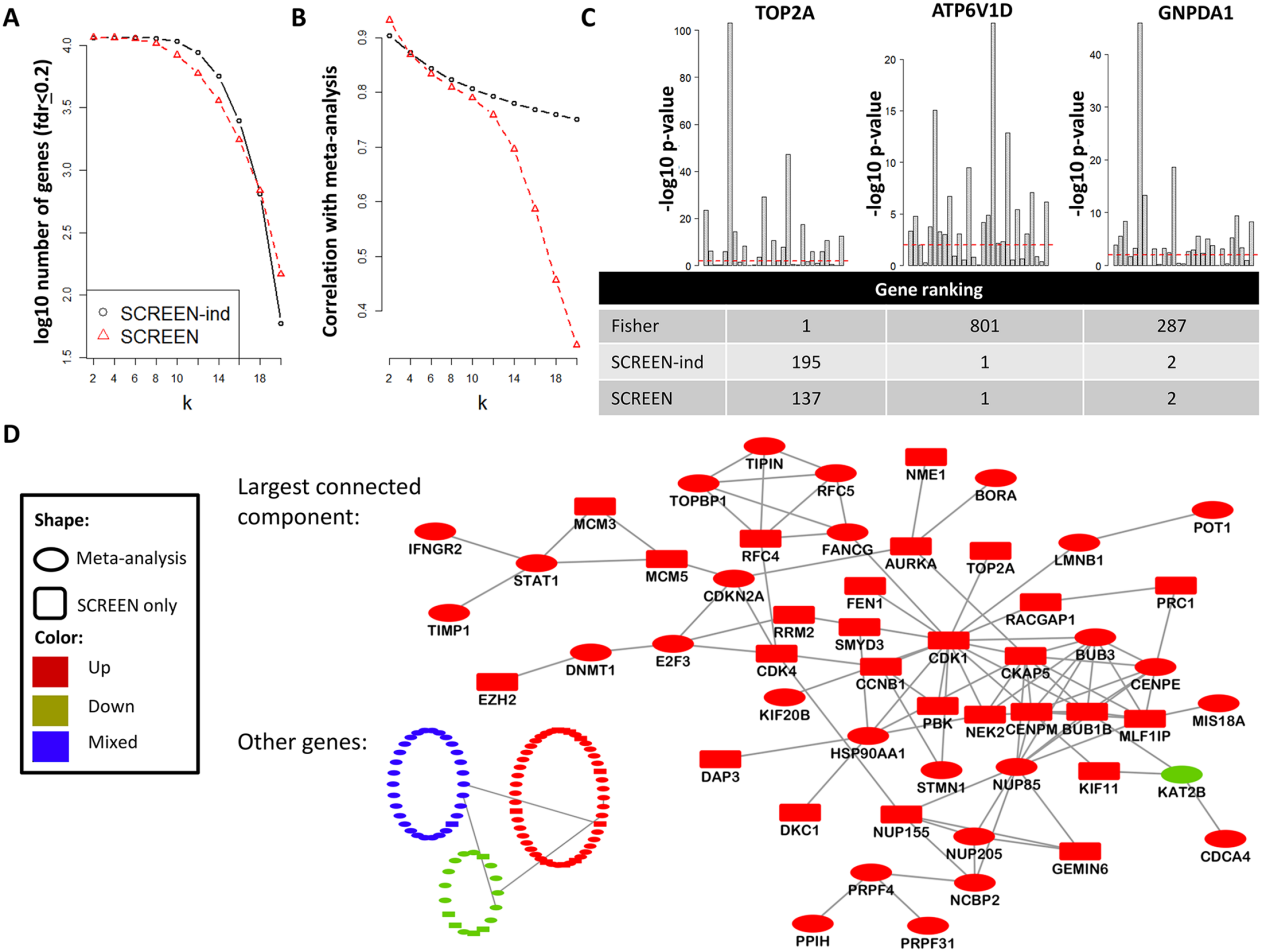
DEG dataset analysis. A) The number of reported genes at 0.2 *fdr* by SCREEN and SCREEN-ind as a function of *k*. B) The Spearman correlation between gene ranking of SCREEN and SCREEN-ind and of Fisher’s meta-analysis as a function of *k*. C) The top ranked genes and their p-values. Top: the p-values of TOP2A, ATP6V1D, and GNPDA1 in each study. Bottom: the rank of these genes according to each of the methods (with k = 20 for SCREEN and SCREEN-ind). ATP6V1D has a very low rank according to Fisher’s meta-analysis even though it has consistently low p-values. D) Network analysis of the 147 genes reported by SCREEN with *k* = 20. Nodes are genes, and edges are either protein-protein interactions or known pathway interactions. The largest connected component is shown in detail, and the rest of the genes and their interactions are shown at the bottom left. For each gene we calculated the number of up- and down-regulated t-statistics with a p-value ≤ 0.01. Genes for which the ratio between the up- and down events was ≥ 3 (≤ 1/3) were considered consistently up-regulated (down-regulated) in cancer (red and green nodes, 99 and 18 genes, respectively). All other genes were considered as mixed (blue nodes, 30 genes). Oval nodes represent genes ranked among the top 200 genes according to Fisher’s meta-analysis (note that even at 10^−5^ Bonferroni correction, more than 10,000 genes were selected in the meta-analysis, We therefore compared to the topmost genes, choosing the number 200 arbitrarily). Rectangular nodes are genes detected only by SCREEN.

Fig 3A shows the number of selected genes identified by SCREEN and SCREEN-ind (at *fdr* 0.2), as a function of *k*, the minimum number of studies on which a gene must be detected. For *k* < 10 the majority of the genes had low *fdr*, suggesting that most genes were differentially expressed in *k* or more studies. For *k* ≤ 17, SCREEN-ind reported more genes than SCREEN. However, SCREEN reported many more genes for higher *k* values. For example, for *k* = 20 SCREEN-ind detected 59 genes, whereas SCREEN detected 147. In addition, for each *k* we compared the output of each algorithm to Fisher’s meta-analysis. Here, we used Spearman correlation to compare the gene ranking obtained by the methods. Fig 3B shows the results as a function of *k*. The correlation for low *k* is near perfect (close to 1 for *k* = 2) and decreases with *k*. Fig 3C depicts three examples of genes with different ranks: TOP2A, ATP6V1D, and GNPDA1. For each gene the plot shows its −*log*_10_ p-value in each study, as well as the rank of the gene according to each of the methods. TOP2A was the top ranked gene in Fisher’s meta-anlaysis, but had much lower ranks in SCREEN-ind and SCREEN. ATP6V1D and GNPDA1, which were the top two genes of SCREEN and SCREEN-ind, respectively, had much lower ranks in Fisher’s meta-analysis. A comparison of the p-value patterns shows that ATP6V1D and GNPDA1 acheived higher rankings even though TOP2A had more extremely low p-values (e.g., <10^−20^). These examples show that SCREEN highlighted genes that were differential consistently across many studies, whereas meta-analysis (as expected) was more sensitive to extremely low p-values. Moreover, the rank-based comparison to Fisher’s method shows that selection of genes in such datasets is not only a question of adjusting a threshold, as the gene ranks are very different.

Our discoveries above were based on genes with consistently low p-values in cancer studies. However, the analysis did not use the direction of the differential expression. To shed more light on the directionality we analyzed the 147 genes detected by SCREEN for *k* = 20. For each gene, we compared the times the reported t-statistic was negative and positive (corresponding to down- and up-regulation, respectively). Genes for which the number of negative values was at least thrice (respectively, at most one third of) the number of positive values were denoted as down-regulated (up-regulated). In total, there were 18 down-regulated and 99 up-regulated genes. The remaining 30 genes were denoted as mixed (see S8B Fig).

Using a network of protein-protein interactions and pathway interactions we plotted the subgraph induced by our 147 genes, see Fig 3D. Interestingly, the largest connected component was composed of 52 up-regulated genes and only a single down-regulated gene (KAT2B). Also, 88 out of 94 edges in the network connected up-regulated genes. Many of the genes in this “active” module were not ranked among the top 200 meta-analysis genes obtained using Fisher’s method, including genes that are well known to play major roles in cancer formation and progression. For example. CDK1 is a master regulator of cell division, and CKAP5 is important for cytoplasmic microtubule elongation and is known to be over-expressed in colonic and hepatic tumors [28–30]. Other important detected genes that were not among the top 200 meta-analysis genes were CDK1, MCM3, and MCM5. The most enriched GO term in the up-regulated gene set was mitotic cell cycle (38 genes, *q* < 10^−28^) and the most enriched term in the active module was spindle organization (10 genes, *q* = 5.7 ⋅ 10^−11^).

In summary, our results show that SCREEN revealed a large gene set of consistently up-regulated genes in cancer that are highly relevant in function. On the other hand, while the results of the meta-analysis were also informative they are very sensitive to genes that achieve extreme p-values in one or a few studies and thus may down-weigh consistency. SCREEN was instrumental for separating the main up-regulated cancer genes that were consistent across most cancers, from other genes that manifested few tissue-specific strong effects.

#### The HLA dataset

Shukla et al. [20] used RNA-seq data to perform differential expression analysis between cancer samples with somatic mutations in the HLA complex and samples without such mutations. The analysis was carried across 11 different TCGA studies, each of a different cancer subtype. The p-value matrix, taken from [20], had 18,128 rows (genes) and 11 columns. Similar to the Cancer DEG dataset, the p-values were based on a one-tail test for differential expression: p-values near zero represent up-regulation, and p-values near 1 represent down-regulation. Fisher’s method was used to assess the overall significance of a gene, and a total of 119 genes were selected using a p-value cutoff of 10^−10^.

We applied both SCREEN and SCREEN-ind on these data. Similarly to the DEG dataset, for *k* ≤ 3 more than 5000 genes had low *fdr*. However, here the number of genes decreased rapidly with *k* so that a few hundred were found for *k* = 4, and only a single gene was reported by SCREEN only for *k* ≥ 5 (S7 Fig). Unlike the DEG dataset, the overall correlation with Fisher’s meta-analysis was lower but the top ranked genes of SCREEN (TNNC2 and IFNG) had also very high ranks in Fisher’s method, see S7 Fig.

For *k* = 4 SCREEN reported many more genes than SCREEN-ind (405 vs. 135) genes, and both methods reported more genes than the original analysis (S7D Fig). When performing functional analysis of the 405 genes detected by SCREEN most genes were consistently up- (154) or down-regulated (199), and only 52 genes were mixed. In pathway enrichment analysis our detected gene sets obtained similar results to the original publication and extended them, see S1 Table. Importantly, we recapitulated the main discovery in [20] of up-regulation of multiple immune related processes. Unlike the original publication, our analysis detected enrichment for cancer related pathways in the down-regulated gene sets of SCREEN, including Wnt signaling (*q* = 0.02) and axon guidance (*q* = 0.04).

**Fig 4:**
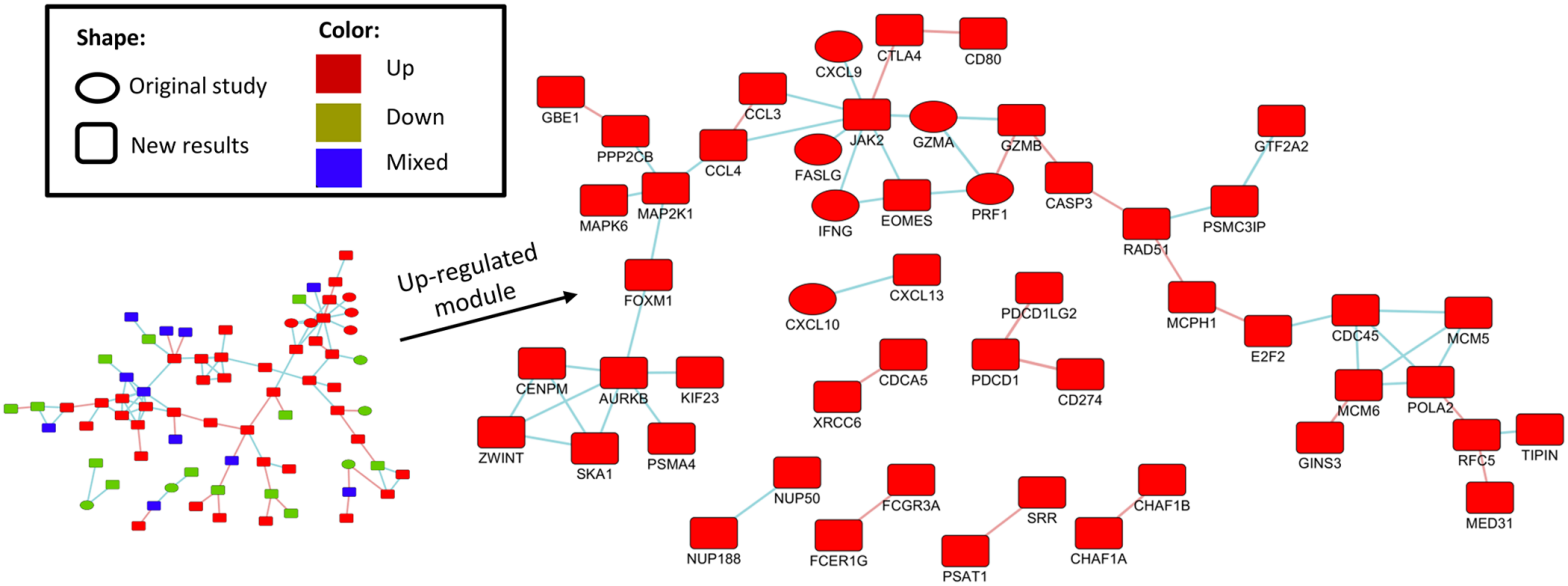
The largest connected component produced by the 405 genes reported by SCREEN in the HLA dataset. Nodes are genes, and edges are either protein-protein interactions or known pathway interactions. Left: all genes in the component, including up-, down-regulated, and mixed genes. Right: the up-regulated genes only. This subnetwork suggests high activity of immune response (which was identified in the original study). Two central genes in the immune response are INFG and JAK2. Our analysis detected both, whereas the original study detected only IFNG. Moreover, the connectivity of the network is established by our newly detected genes.

Fig 4 shows the largest connected component in the network induced by the up-regulated gene set. The network contains a few genes reported in the original study and many newly reported genes, which connect them into a single component. Unlike the DEG dataset, the main connected component contains both up-, down-, and mixed-regulation patterns, but the up-regulated genes form the backbone of the component and are responsible to most of the connecting links. By focusing on the up-regulated genes we detected an active module of genes involved in activation of immune response, anti-tumor activity, and T-cell activation. Many high degree nodes in the module, such as JAK2, were not reported in the original study. In summary, our results provide a comprehensive picture of the molecular response in patients with HLA mutations. SCREEN recapitulated the main findings obtained in the original study and revealed novel ones.

## Discussion

We presented here several novel algorithms for detecting replicated associations using an empirical Bayes approach. Our main algorithm, called SCREEN, outperformed other approaches in many scenarios and had consistently low false discovery proportion and high true discovery rates in all simulations.

SCREEN works in three stages. First, it clusters the studies based on their pairwise correlations, which are learned via EM. Second, it performs replicability analysis within each cluster using our restricted EM approach. This method goes beyond previous studies by restricting the possible number of study configurations that are kept in memory. As a result, the method can analyze large study clusters, by computing an upper bound for the *fdr* instead of an exact estimation. Finally, the results of the replicability analyses of the clusters are merged using dynamic programming. For a given *k*, the output of SCREEN is the *fdr*_*k*_ value for each gene, which can be used to detect genes that are non-null in at least *k* studies.

We have shown that SCREEN performs well on various simulated scenarios, as well as on real datasets. Specifically, we analyzed two collections of cancer-related gene expression studies. In both cases the discovered gene sets highlighted active gene modules with pertinent functions.Such modules are revealed by the projection of the discovered genes on an interaction network and focusing on well-connected subnetworks. Notably, standard meta-analysis does not reveal many of the genes and generates more fragmented and less coherent subnetworks. For example, in the cancer DEG datasets, some of these genes are central in the network and are known master regulators of cell cycle (e.g., CDK1). In summary, we demonstrated replicability analysis as a standard tool for analyzing a large collection of studies, and provided novel algorithms that are accurate and scalable.

While the current version of SCREEN does not model the direction of the statistic directly (i.e., up- or down-regulation), we addressed this point empirically in our examples and showed that most genes were consistent in their direction. For the sake of functional analysis we required a gene to have the same direction in at least 75% of the studies. This threshold reflects a reasonable selection between the number of reported genes and the consistency requirement (see S8 Fig). Of course, users can change this threshold to require a higher (or lower) consistency in direction in specific applications. Also, note that detection of “mixed sign” genes is an important feature of our analysis: the causality of such genes is questioned as they likely represent downstream effects.

The strategy of SCREEN can be extended to a more complex definition of replicability across study clusters. For example, a researcher may seek genes that are replicable across one or more study clusters, where a gene is replicable in a cluster only if it is non-null in at least some predefined percentage of the studies in that cluster. See S1 Text for a discussion on this topic.

A variety of methods for integration of different studies have been developed in GWAS but their goals are different from SCREEN’s. These methods typically assume a standard meta-analysis null hypothesis that the effect size is zero across all studies [31]. As such, they do not address the question of replication directly even if a clustering of the studies is considered [32]. Moreover, some of the Bayesian approaches that were developed for GWAS utilize a subjective prior and do not estimate it form the data [33]. Finally, as we have shown, SCREEN outperforms repfdr, which was originally developed for GWAS data [9].

Our study has some limitations that can be addressed in future research. First, we assumed that genes are independent. This assumption is usually made by state of the art methods, but is often incorrect. In our case, it was used to obtain tractable algorithms (both the dynamic programming and the EM). Second, while our algorithms report *fdr*_*k*_ values of genes, we currently do not estimate their variance. Third, selection of *k* was done manually on real datasets based on the specific biological question and the number of reported genes (e.g., Fig 3A).

Fourth, our restricted EM approach to analyze study clusters is a heuristic that only guarantees convergence into a local optimum. Thus, while our algorithm has a deterministic starting point for the EM, setting starting points at random may change the obtained upper bounds for the *fdr*. Indeed, when we tried using random starting points, the set of reported genes in the cancer DEG dataset changed (by up to 50 genes, but still leaving over 80 genes from the original results), and no change was observed in the HLA dataset. Another important aspect of the heuristic is the number of allowed gene configurations. As an example, SCREEN with *n*_*H*_ = 10000 configurations on the cancer DEG dataset finds more than 100 additional genes for k = 20, whereas the *fdr* of the genes reported in our original analysis (Fig 4) is kept low, illustrating that the results are consistent (see S9 Fig).

Fifth, while our simple Exp-count approach to estimate the expected number of non-null realizations of a gene performed reasonably well in some simulated scenarios, it is only partially justified theoretically (see S1 Text). Finally, our methods rely on fixed estimates of the two-groups model of each study. Future methods could go a step further and estimate all parameters (i.e., both the study parameters and the gene configuration probabilities) in a single flow.

## Materials and methods

### Learning a two-groups model in each study

We tried two implementations of two-groups estimation algorithms. The first *locfdr* [22], provides two options to learn the empirical null: maximum likelihood and central matching. By default, we used the maximum likelihood estimator. However, in practice this algorithm might converge to a solution in which ${\hat{\pi }}_{0}>1$. Whenever this occured, we tried the central matching approach instead. If the new estimator also had ${\hat{\pi }}_{0}>1$ we used the theoretical null.

The second approach was based on two previous methods: *Znormix* [34] and *fdrtool* [35]. Znormix uses EM to learn a mixture of Gaussians, whereas *fdrtool* assumes that the null distribution is a half normal distribution. Here, we applied an EM approach to the absolute values of the z-scores. We extended these methods by learning a mixture of a half normal with *σ* ≥ 1 for the null distribution, and a normal distribution with *μ* > 0 for the non-nulls. We call this approach *normix*.

In practice, we discovered that our EM algorithm is sensitive to high values in the estimation of *f*_1_. In addition, the methods above do not exploit additional information that could be obtained from the two-groups model: an estimation for the power of a study [19]. That is, this is a measure of how separated the two groups are. In our analyses, we took a very stringent approach: in each study we multiply *f*_1_(*z*) by the estimated power of that study. The effect is a shrinkage in the *f*_1_(*z*) values that is proportional to the estimated quality of the study.

### Clustering the studies

SCREEN relies on a known partition of the studies into clusters. In this section we use an empirical Bayes approach to obtain the clusters. Our analysis has two main parts: learning a network, and clustering.

First, we create a correlation network among the studies. For each study *i*, let *a*_*i*_ = *P*(*h*_*i*_ = 1) be the marginal non-null probability in that study. For studies *i*, *j* let *a*_*i*,*j*_ = *P*(*h*_*i*_ = 1 ∧ *h*_*j*_ = 1) be the shared non-null probability of the two studies. We estimate these parameters as follows: *a*_*i*_ and *a*_*j*_ are taken from the two-groups model of each study, and *a*_*i*,*j*_ is estimated by running our EM approach on the data of these two studies. The correlation of the studies is then estimated by:

$$\begin{array}{c} r_{i,j}=\frac{a_{i,j}-a_{i}a_{j}}{\sqrt{a_{i}\left(1-a_{i}\right)a_{j}\left(1-a_{j}\right)}} \end{array}$$

We obtain a robust estimation of *r*_*i*,*j*_ by taking the mean of 100 bootstrap runs of the procedure above. That is, in each run we reestimate *a*_*i*,*j*_ by running the EM on a bootstrap sample of the genes (*n*/2 genes out of *n*, sampled with replacement).

Next, we cluster the network using the infomap algorithm [36]. Here, communities are detected using random walks in the underlying graph. As the input for this algorithm is an unweighted network, we used a threshold of 0.1 for the absolute correlation of study pairs to determine edge presence. This threshold is relatively low for general clustering tasks as it does not guarantee high homogeneity within clusters. However, it guarantees that the clusters discovered by SCREEN will be well-separated. In practice, our clustering approach found the correct clustering of studies in all simulations performed.

### Other multi-study analyses

In order to evaluate our *fdr* approaches we compared them to several extant methods for multi-study analysis. Here we outline them briefly.

#### Fisher’s meta-analysis

We used Fisher’s meta-analysis to merge the p-values of each gene into a single p-value. We then applied the BH FDR algorithm to account for multiple testing. Note that Fisher’s meta-analysis is not meant for replicability analysis and it does not take *k* as input. Nevertheless, we added it to the comparison due to its use in recent publications (e.g., [20, 37, 38]).

#### Counting-based BH analysis

Here we run the BH multiple testing correction algorithm in each study separately. For each gene we count the number of q-values lower than some predefined threshold. In this study we used a threshold of 0.1. We call this method BH-count.

#### Counting-based tdr analysis

In this analysis, for each gene, we use the local true discovery rates (tdr) values obtained from the marginal two-groups model for each study. We then sum over these rates for the gene:

$$\begin{array}{c} \sum_{j=1}^{m}\left(1-P\left(h_{i,j}=0|Z_{i,j}\right)\right) \end{array}$$

This statistic can be interpreted as a biased estimator for the expected number of non-null realizations of gene *i*. See the S1 Text for more details. We call this method Exp-count.

### Enrichment and network analysis

Network analysis and visualization was done in Cytoscape [39]. The GeneMANIA Cytoscape app [40, 41] was used to create the gene networks of the selected gene sets. GO enrichment analysis was performed using Expander [42].

### Availability

The datasets and an implementation of SCREEN are available in S1 Data.

## Supporting information

S1 Fig Simulation results: 20 independent studies with *β*(1,1000) non-null p-values. A) locfdr, B) normix. The left column shows the empirical FDP. The right column shows the Jaccard scores only for methods that had a consistently low FDP values (< 0.2) in each case and for all *k* values. These scores are calculated by comparing the output gene set of each method for each *k* to the set of genes for which the real number of non-nulls was at least *k*. SCREEN and SCREEN-ind (SCRN-ind) have identical results and achieve the top or almost top Jaccard for *k* ≤ 4.
(PDF)

S2 Fig Simulation results using locfdr and non-null distribution of *β*(1,100). A) 20 independent studies. B) Four clusters of 10 studies each with a relatively low correlation within each cluster. C) Four clusters of 10 studies each with a relatively high correlation within each cluster. The left column shows the empirical FDP. The right column shows the Jaccard scores only for methods that had a consistently low FDP values (< 0.2) in each case and for all *k* values. BH-count, SCREEN and SCREEN-ind (SCRN-ind for short) are the only methods that are shown on the right in all cases. BH-count had very low Jaccard scores. In A) SCREEN-ind had superior results. In B-C) Except for *k* = 2, SCREEN had similar or better performance compared to SCREEN-ind.
(PDF)

S3 Fig Simulation results: 20 independent studies with *β*(1,10) non-null p-values. A) locfdr, B) normix. The left column shows the empirical FDP. The right column shows the Jaccard scores only for methods that had a consistently low FDP values (< 0.2) in each case and for all *k* values. These scores are calculated by comparing the output gene set of each method for each *k* to the set of genes for which the real number of non-nulls was at least *k*. The Jaccard scores here are always very low, and the FDP scores of SCREEN and repfdr-UB might be high. However, when the FDP scores are high, very few genes are reported by SCREEN (≤ 4), whereas repfdr-UB might report ≥ 10 genes. Note that the scale of the Jaccard plots was cleaved to show the very low values.
(PDF)

S4 Fig P-value histograms of all studies in the cancer DEG dataset. (PDF)

S5 Fig P-value histograms of all studies in the HLA dataset. (PDF)

S6 Fig Inferred correlation networks among the DEG and HLA studies. Left: inferred correlations. Right: binary correlation. Each point represents correlation with absolute value ≥ 0.1. Study names are colored by their cluster assignment.
(PDF)

S7 Fig HLA dataset analysis. A) The number of reported genes at 0.2 *fdr* by SCREEN and SCREEN-ind as a function of *k*. B) The Spearman correlation between gene ranking of SCREEN and SCREEN-ind and of Fisher’s meta-analysis as a function of *k*. C) The top ranked genes and their p-value in each study. Top: the p-values of TNNC2 and IFNG. Bottom: the rank of these genes according to each of the methods (with k = 4 for SCREEN and SCREEN-ind). Both genes are highly ranked by all methods. D) The number of genes reported by each method.
(JPG)

S8 Fig Differential expression direction analysis of the reported genes. For each reported gene the fraction of datasets with a positive t-statistic is shown (out of the studies with *p* < 0.01). Genes are ordered by the fraction. Red: genes up-regulated in 75% of the studies. Green: genes down-regulated in 75% of the studies. Blue: all other genes. A) HLA dataset, *k* = 4. B) Cancer DEG dataset, *k* = 20.
(PDF)

S9 Fig Effect of increasing the number of allowed configurations within SCREEN. SCREEN was run with *n*_*H*_ = 10000 allowed configurations. The number of detected genes for *k* = 20 more than doubled. While new genes are detected, the genes reported using *n*_*H*_ = 1024 only (as in the main text) remain significant (except for 5 genes). The figure shows the distribution of local fdr values for all genes discovered on the cancer DEG dataset with *n*_*H*_ = 10000, split into those that were also significant using *n*_*H*_ = 1024 and the rest.
(PDF)

S1 Text (PDF)

S1 Table The studies of the cancer DEG dataset. (PDF)

S1 Data A zip file with the code of all methods and the matrices of the real datasets. (ZIP)
