# Supplementary figures and images for: Extracting replicable associations across multiple studies: Empirical Bayes algorithms for controlling the false discovery rate
Source: PLoS Comput Biol. 2017 Aug 18;13(8):e1005700. doi: 10.1371/journal.pcbi.1005700 (PMC5576761; doi:10.1371/journal.pcbi.1005700)

### A) 20 indep studies

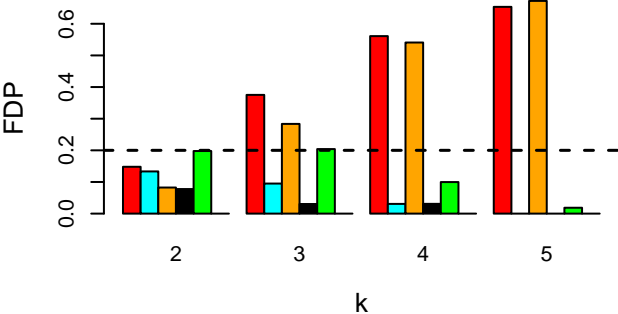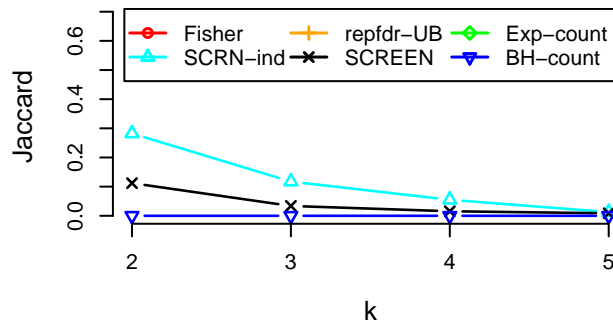

### B) 4 clusters of 10 studies, $r=0.4$

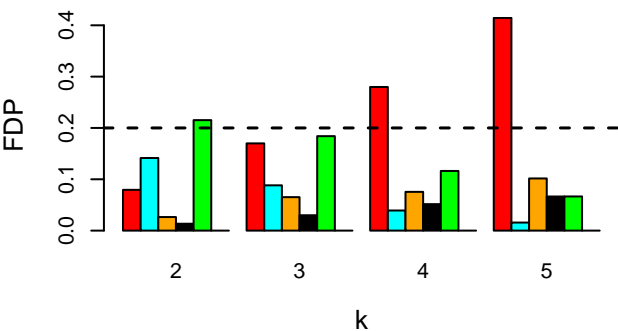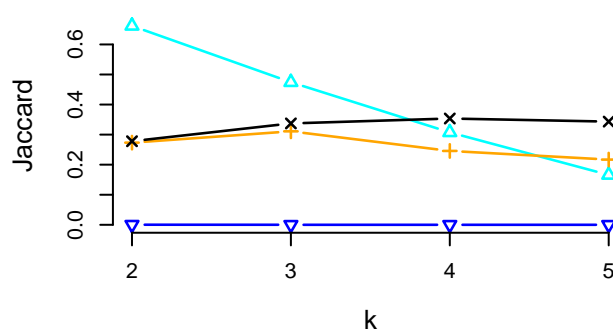

### C) 4 clusters of 10 studies, $r=0.8$

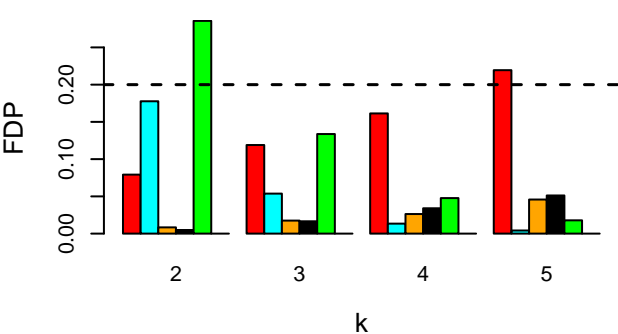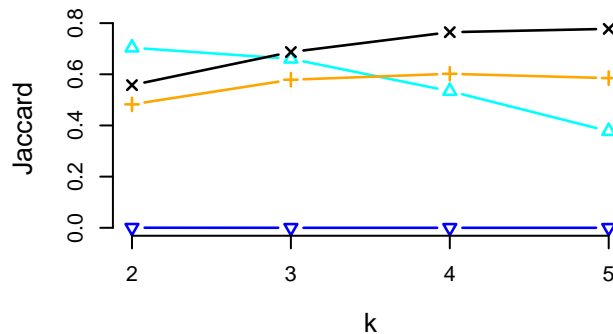

Supplement: S1 Fig — A) locfdr, B) normix. The left column shows the empirical FDP. The right column shows the Jaccard scores only for methods that had a consistently low FDP values (< 0.2) in each case and for all k values. These scores are calculated by comparing the output gene set of each method for each k to the set of genes for which the real number of non-nulls was at least k. SCREEN and SCREEN-ind (SCRN-ind) have identical results and achieve the top or almost top Jaccard for k ≤ 4. (PDF) [file pcbi.1005700.s001.pdf]

**A) f1=Beta (1,1000), locfdr**

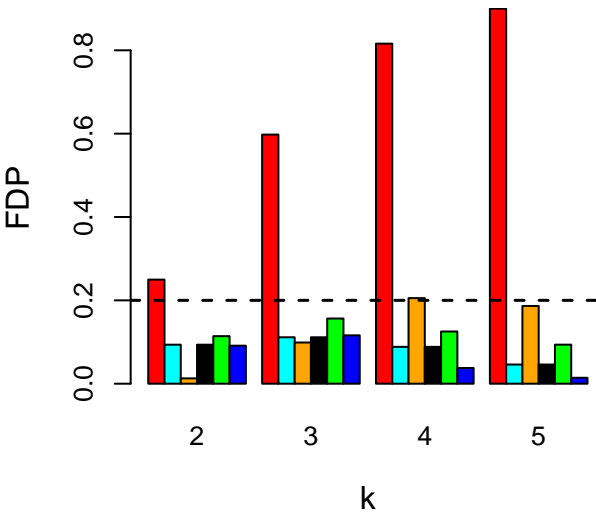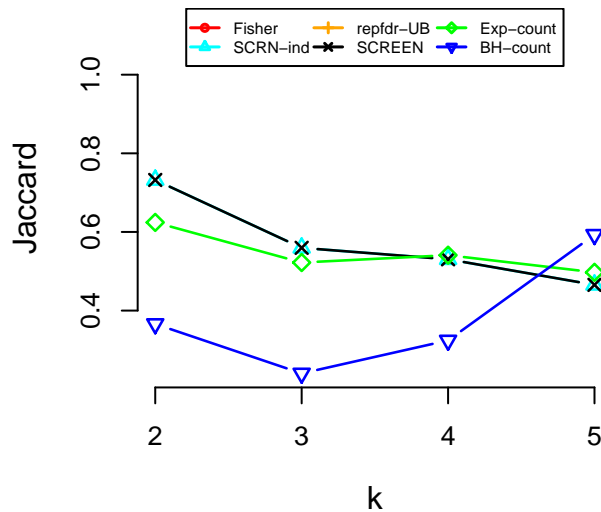

**B) f1=Beta (1,1000), normix**

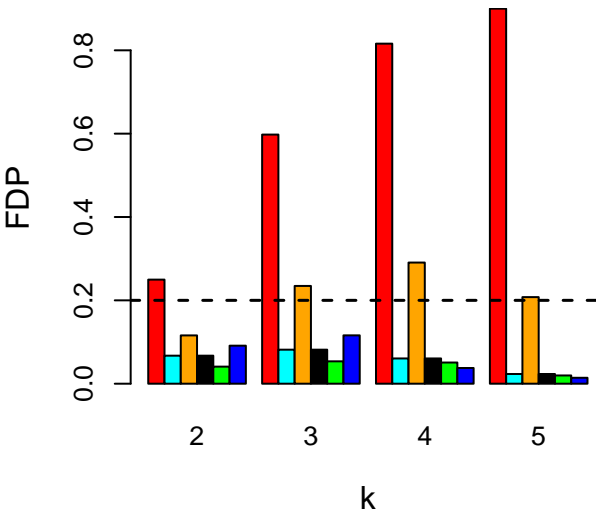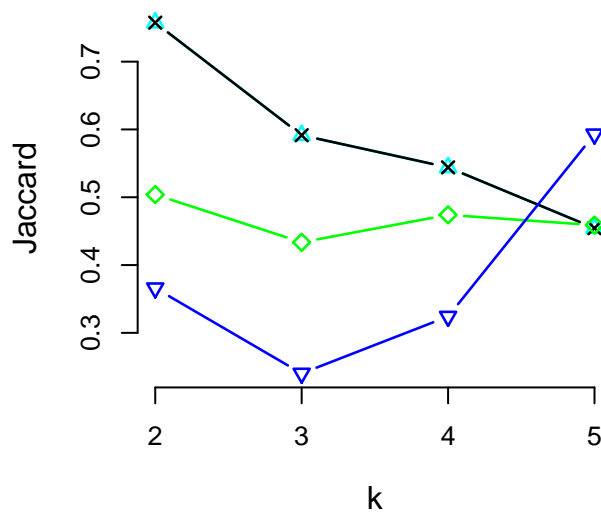

Supplement: S2 Fig — A) 20 independent studies. B) Four clusters of 10 studies each with a relatively low correlation within each cluster. C) Four clusters of 10 studies each with a relatively high correlation within each cluster. The left column shows the empirical FDP. The right column shows the Jaccard scores only for methods that had a consistently low FDP values (< 0.2) in each case and for all k values. BH-count, SCREEN and SCREEN-ind (SCRN-ind for short) are the only methods that are shown on the right in all cases. BH-count had very low Jaccard scores. In A) SCREEN-ind had superior results. In B-C) Except for k = 2, SCREEN had similar or better performance compared to SCREEN-ind. (PDF) [file pcbi.1005700.s002.pdf]

**A)  $f1=\text{Beta}(1,10)$ , locfdr**

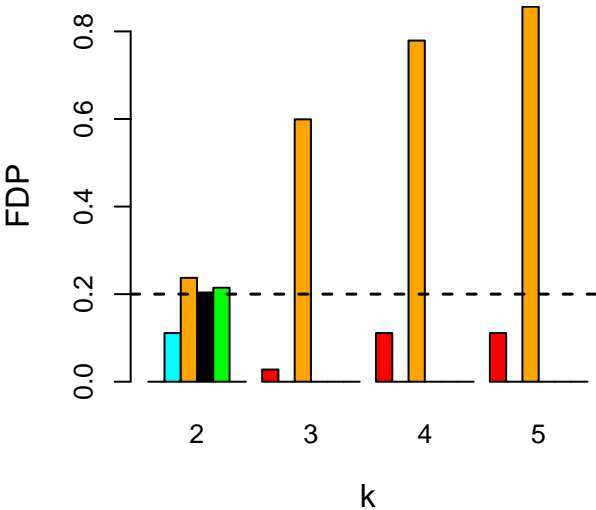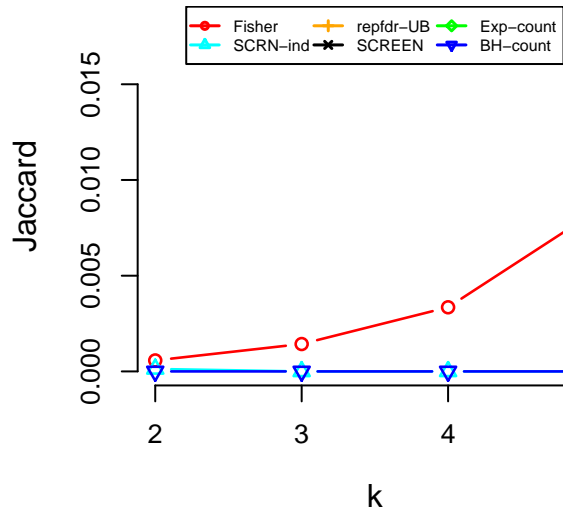

**B)  $f1=\text{Beta}(1,10)$ , normix**

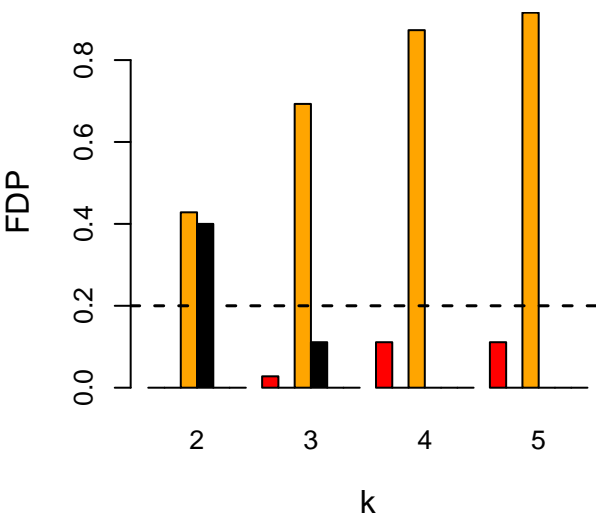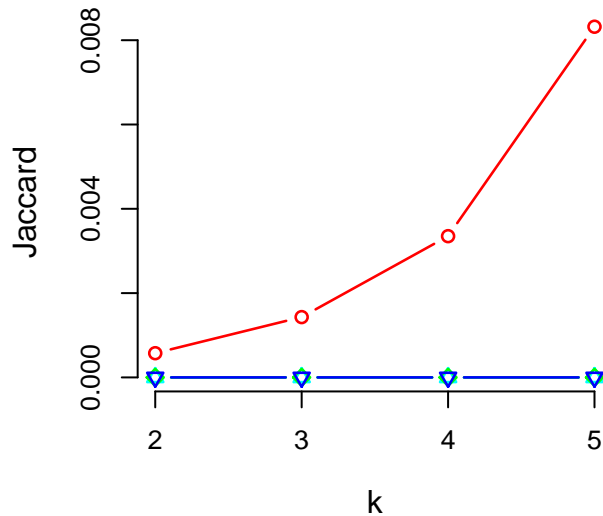

Supplement: S3 Fig — A) locfdr, B) normix. The left column shows the empirical FDP. The right column shows the Jaccard scores only for methods that had a consistently low FDP values (< 0.2) in each case and for all k values. These scores are calculated by comparing the output gene set of each method for each k to the set of genes for which the real number of non-nulls was at least k. The Jaccard scores here are always very low, and the FDP scores of SCREEN and repfdr-UB might be high. However, when the FDP scores are high, very few genes are reported by SCREEN (≤ 4), whereas repfdr-UB might report ≥ 10 genes. Note that the scale of the Jaccard plots was cleaved to show the very low values. (PDF) [file pcbi.1005700.s003.pdf]

**GSE10072**

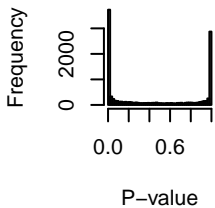

**GSE12452**

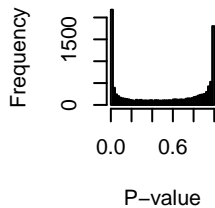

**GSE12453**

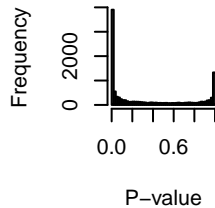

**GSE14245**

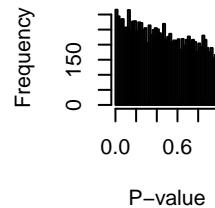

**GSE14407**

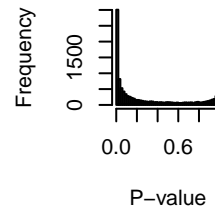**GSE14520**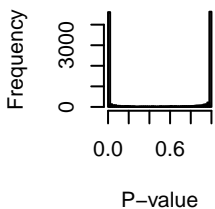

**GSE19804**

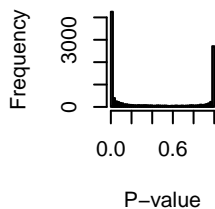

**GSE20189**

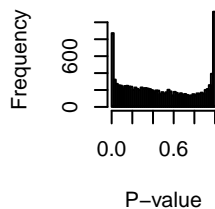

**GSE20347**

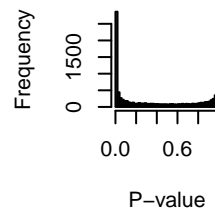

**GSE20437**

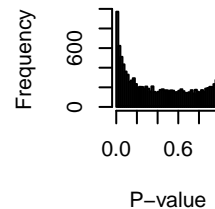

**GSE22529**

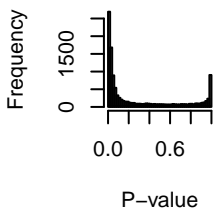

**GSE2549**

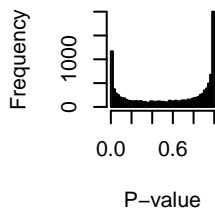

**GSE26566**

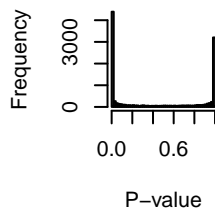

**GSE26910**

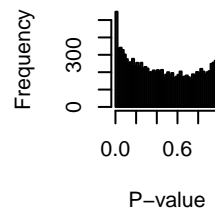

**GSE2719**

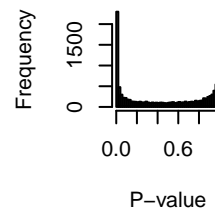

**GSE27562**

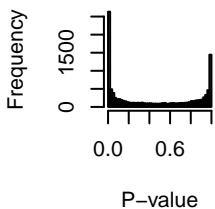

**GSE28735**

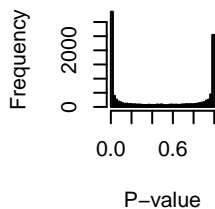

**GSE32665**

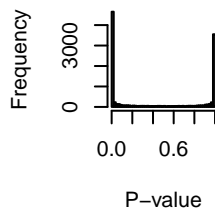

**GSE4107**

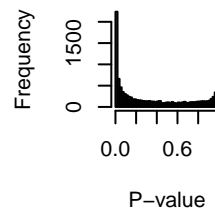

**GSE4115**

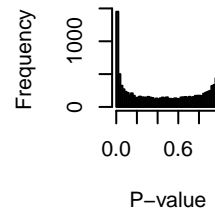

Supplement: S4 Fig — (PDF) [file pcbi.1005700.s004.pdf]

**BLCA**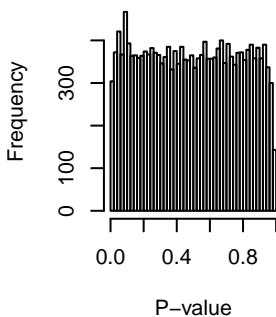**BRCA**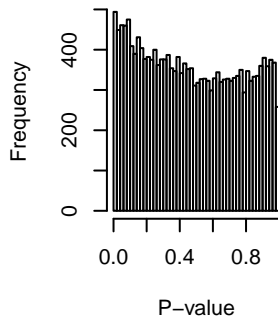**CESC**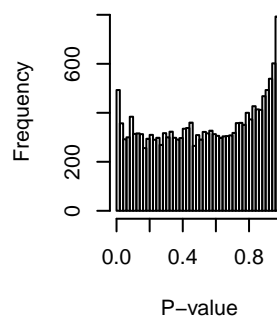**CRC**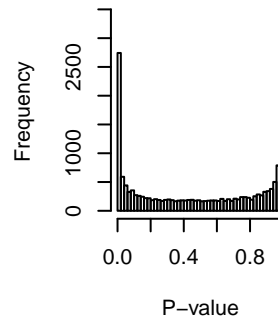**HNSC**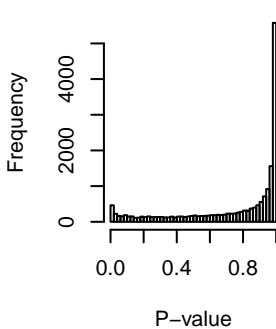**KIRP**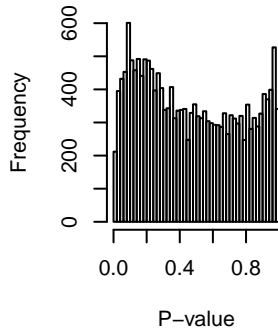**LGG**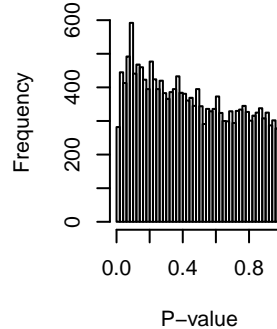**LUAD**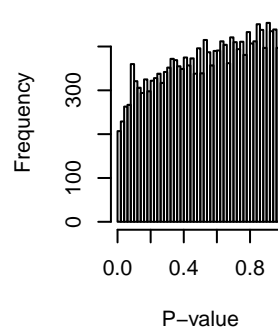**LUSC**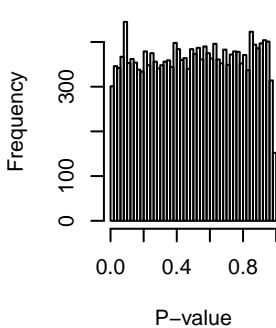**STAD**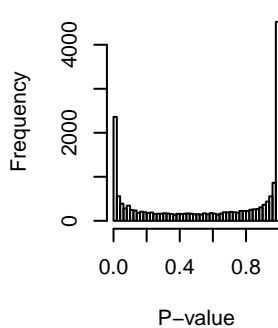**UCEC**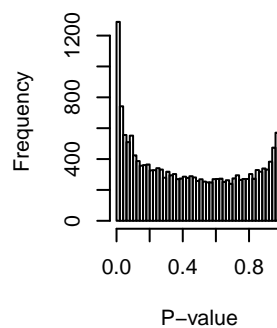

Supplement: S5 Fig — (PDF) [file pcbi.1005700.s005.pdf]

DEG :  $r_{i,j}$

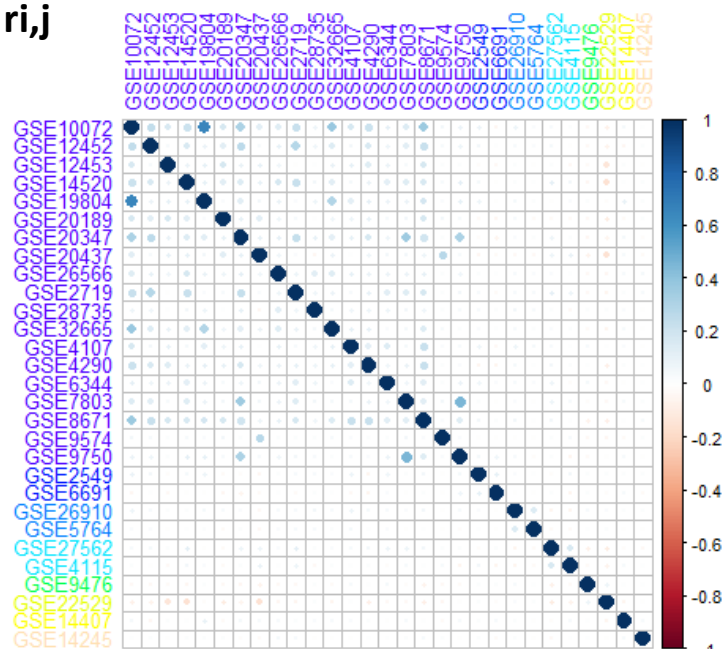

DEG:  $r_{i,j} \geq 1$

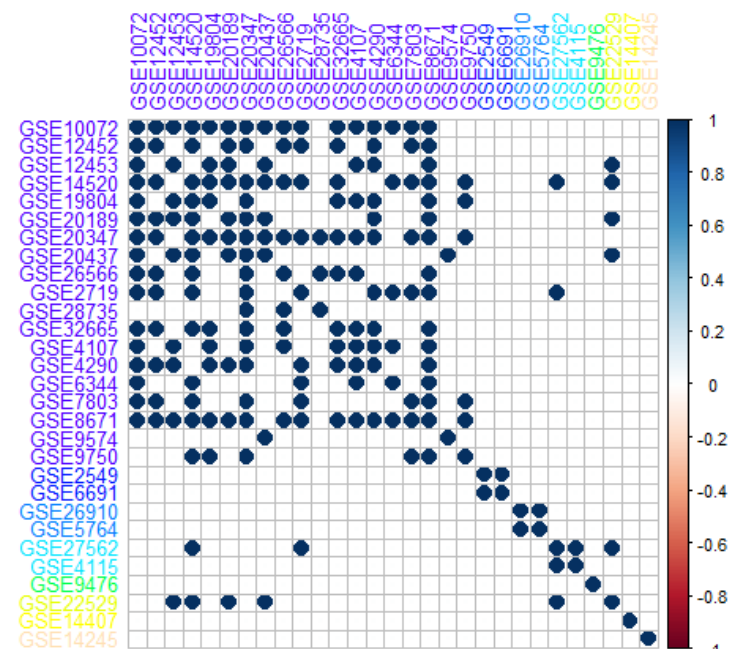

HLA:  $r_{i,j}$

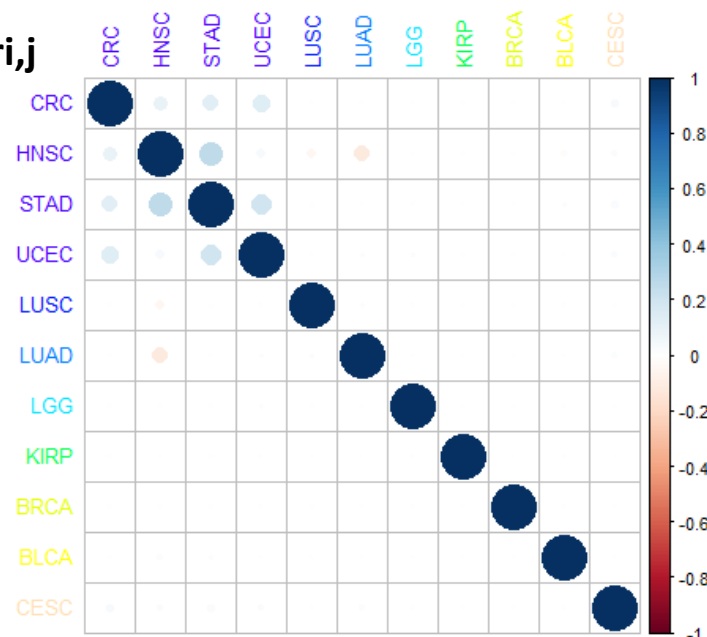

HLA:  $r_{i,j} \geq 1$

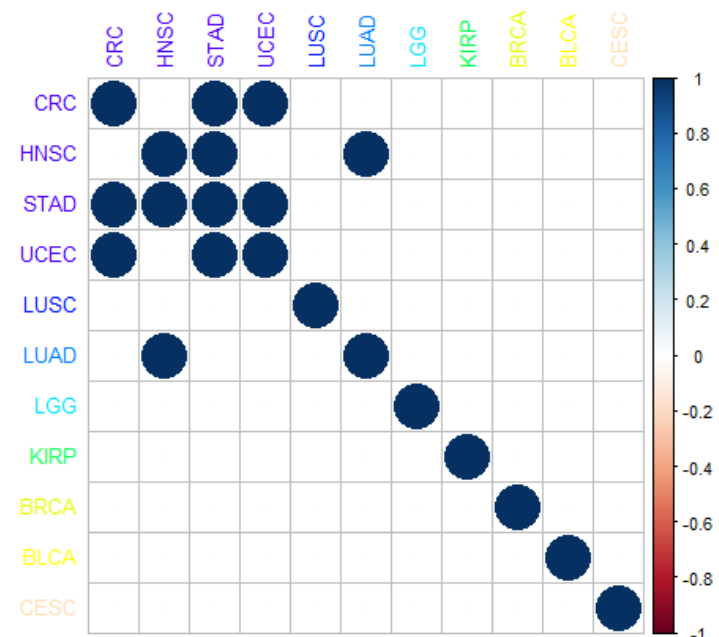

Supplement: S6 Fig — Left: inferred correlations. Right: binary correlation. Each point represents correlation with absolute value ≥ 0.1. Study names are colored by their cluster assignment. (PDF) [file pcbi.1005700.s006.pdf]

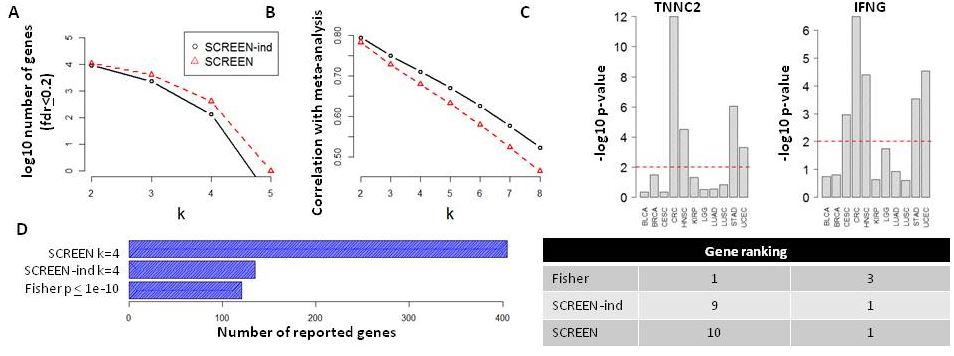

Supplement: S7 Fig — A) The number of reported genes at 0.2 fdr by SCREEN and SCREEN-ind as a function of k. B) The Spearman correlation between gene ranking of SCREEN and SCREEN-ind and of Fisher’s meta-analysis as a function of k. C) The top ranked genes and their p-value in each study. Top: the p-values of TNNC2 and IFNG. Bottom: the rank of these genes according to each of the methods (with k = 4 for SCREEN and SCREEN-ind). Both genes are highly ranked by all methods. D) The number of genes reported by each method. (JPG) [file pcbi.1005700.s007.jpg]

A: HLA dataset

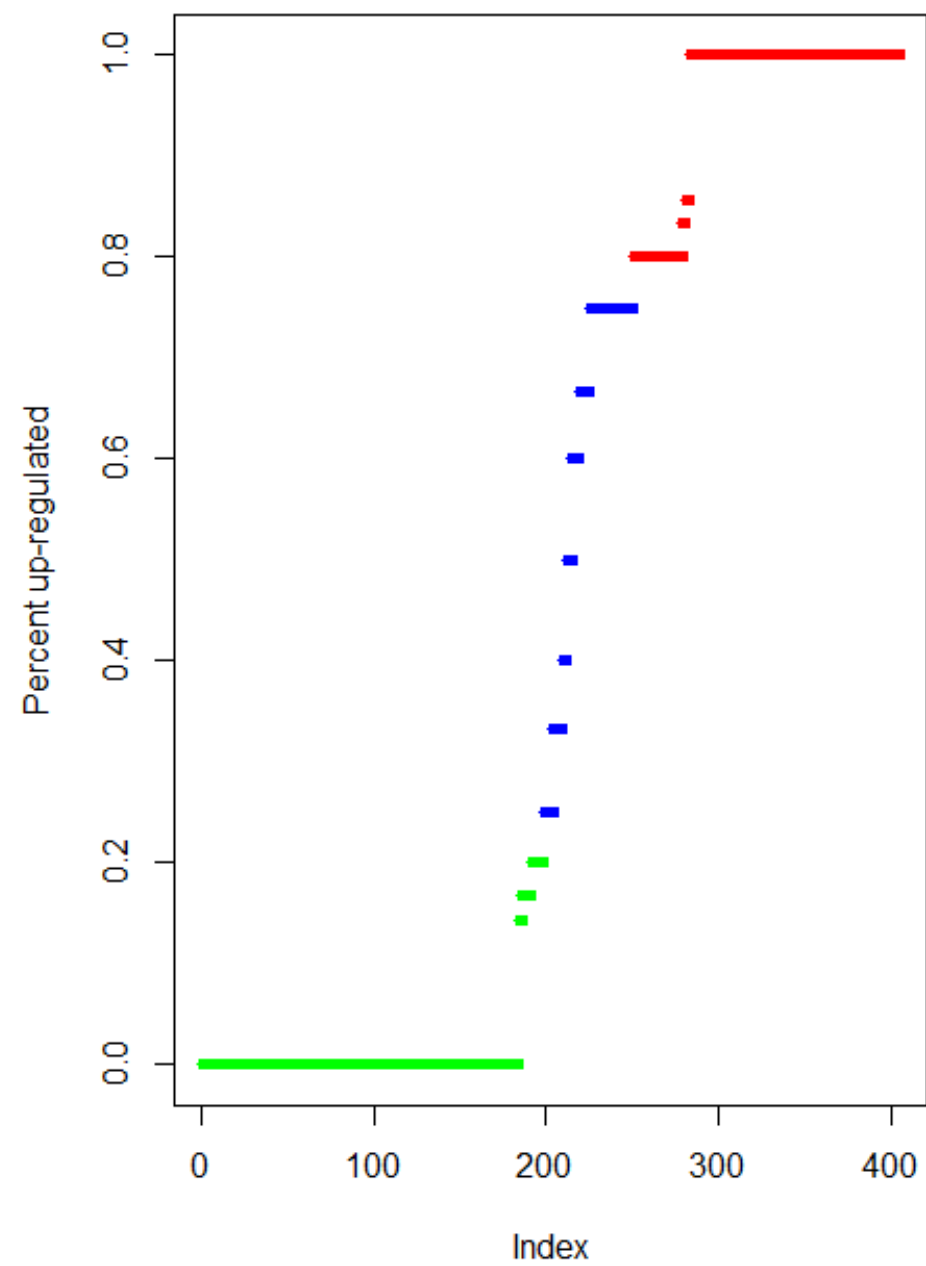

B: Cancer DEG dataset

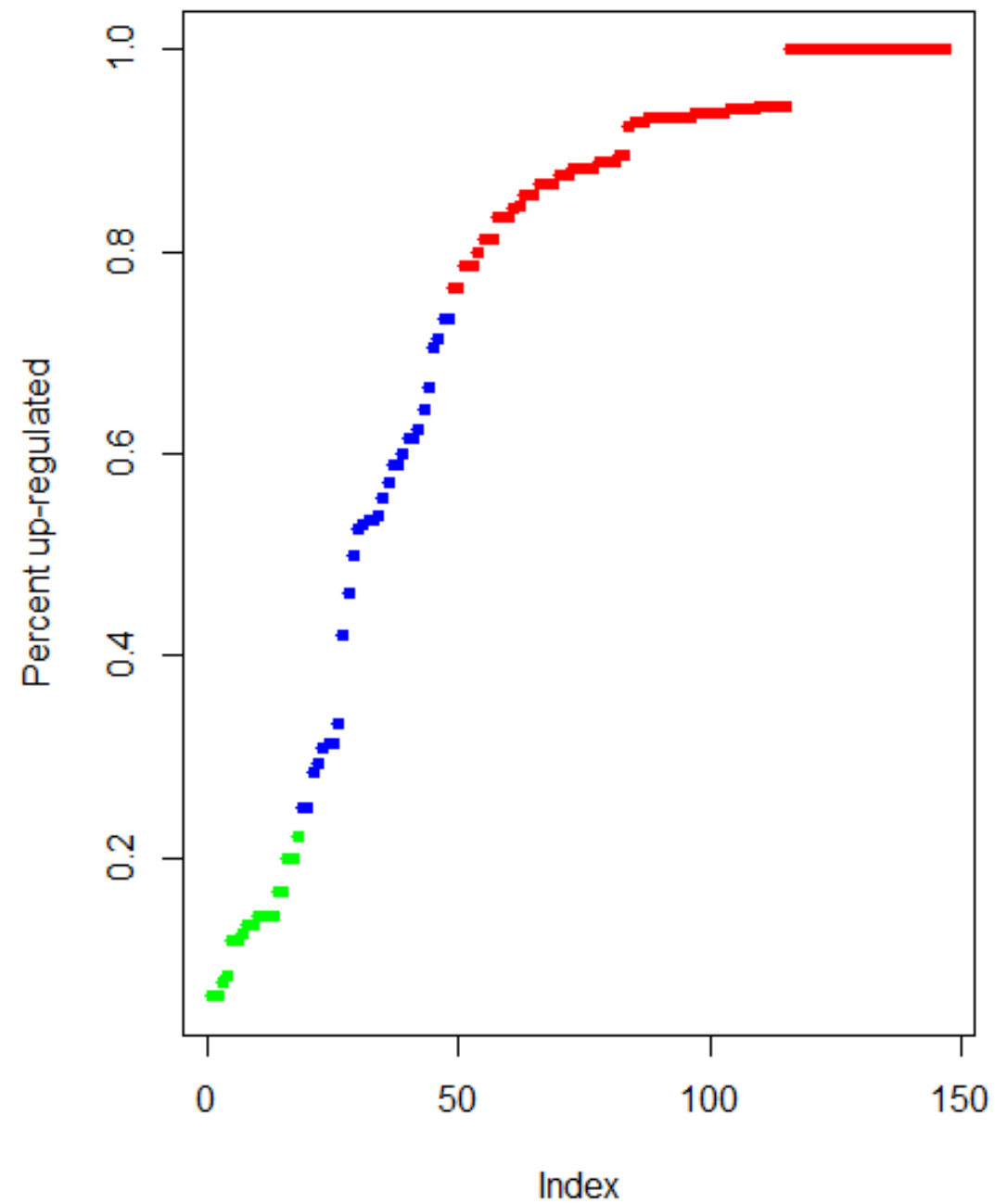

Supplement: S8 Fig — For each reported gene the fraction of datasets with a positive t-statistic is shown (out of the studies with p < 0.01). Genes are ordered by the fraction. Red: genes up-regulated in 75% of the studies. Green: genes down-regulated in 75% of the studies. Blue: all other genes. A) HLA dataset, k = 4. B) Cancer DEG dataset, k = 20. (PDF) [file pcbi.1005700.s008.pdf]

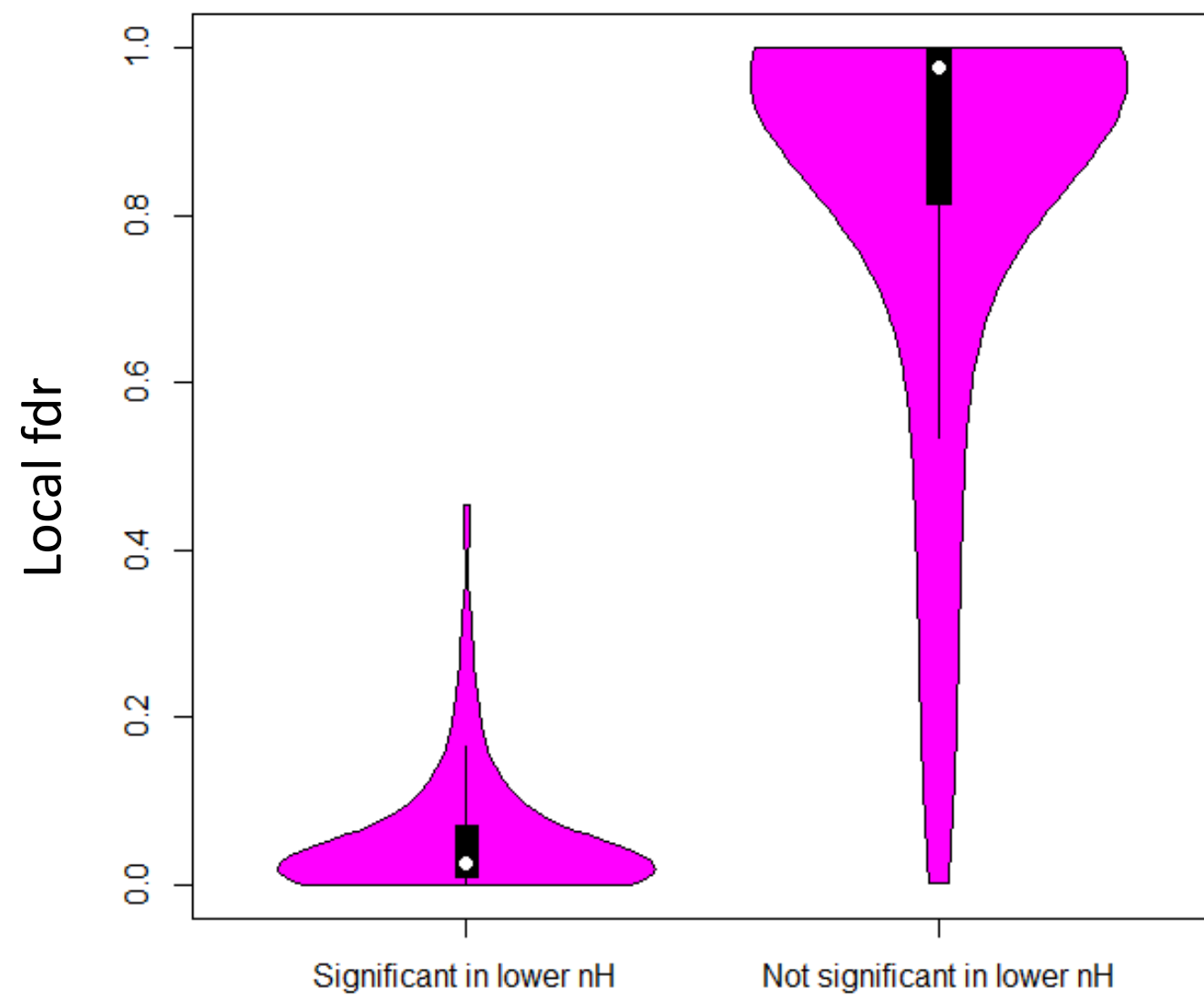

Supplement: S9 Fig — SCREEN was run with nH = 10000 allowed configurations. The number of detected genes for k = 20 more than doubled. While new genes are detected, the genes reported using nH = 1024 only (as in the main text) remain significant (except for 5 genes). The figure shows the distribution of local fdr values for all genes discovered on the cancer DEG dataset with nH = 10000, split into those that were also significant using nH = 1024 and the rest. (PDF) [file pcbi.1005700.s009.pdf]
