# Supplementary material for: Extracting replicable associations across multiple studies: Empirical Bayes algorithms for controlling the false discovery rate
Source: PLoS Comput Biol. 2017 Aug 18;13(8):e1005700. doi: 10.1371/journal.pcbi.1005700 (PMC5576761; doi:10.1371/journal.pcbi.1005700)
Supplement: S1 Text — (PDF) [file pcbi.1005700.s010.pdf]

Supplementary Text

Overview of SCREEN

**Algorithm:** SCREEN
1 Apply the two-groups model on each study
2 Learn pairwise study correlations
3 Cluster the studies
4 For each cluster use the EM algorithm (restricted, if needed) to compute fdr_k
5 Combine the results of clusters using dynamic programming

The expected number of non-nulls of a gene

Let us develop the expected number of non-bull values for gene i.

$$E\left[\sum_{j=1}^{m} H_{i,j} \middle| Z_{i,\cdot}\right] = \sum_{j=1}^{m} E\left[H_{i,j} \middle| Z_{i,\cdot}\right] = \sum_{j=1}^{m} P\left[H_{i,j} = 1 \middle| Z_{i,\cdot}\right]$$

$$= \sum_{j=1}^{m} \frac{P(H_{i,j} = 1) P(Z_{i,\cdot} | H_{i,j} = 1)}{P(Z_{i,j})}$$

For each j we now apply $P(Z_{i,\cdot}) = P(Z_{i,j})P(Z_{i,-j}|Z_{i,j})$ and $P(Z_{i,\cdot}|H_{i,j} = 1) = P(Z_{i,j}|H_{i,j} = 1)P(Z_{i,-j}|H_{i,j} = 1, Z_{i,j})$, to partition $Z_{i,\cdot}$ into $Z_{i,j}$ and the remaining vector $Z_{i,-j}$. This produces the following form for the expectation:

$$E\left[\sum_{j=1}^{m} H_{i,j} \middle| Z_{i,\cdot}\right] = \sum_{j=1}^{m} \left(\frac{P(H_{i,j} = 1)P(Z_{i,j}|H_{i,j} = 1)}{P(Z_{i,j})}\right) \left(\frac{P(Z_{i,-j}|H_{i,j} = 1, Z_{i,j})}{P(Z_{i,-j}|Z_{i,j})}\right)$$

$$= \sum_{j=1}^{m} (tdr_j(Z_{i,j})) \left(\frac{P(Z_{i,-j}|H_{i,j} = 1)}{P(Z_{i,-j}|Z_{i,j})}\right)$$

The term above represents the expectation of non-nulls as a weighted sum over the true discovery rates of gene i in each study j. The weight $\frac{P(Z_{i,-j}|H_{i,j}=1)}{P(Z_{i,-j}|Z_{i,j})}$ can be interpreted as a measure of discrepancy between observing that $H_{i,j} = 1$ and observing $Z_{i,j}$. For example, if all studies are independent the weights are all 1 and we get the sum of the tdr values. On the other hand, if all studies are highly correlated, the values in $Z_{i,-j}$ are very high (say > 5), and $Z_{i,j} = 0$, then $P(Z_{i,-j}|H_{i,j} = 1) > P(Z_{i,-j}|Z_{i,j})$ and the weight of study j will be > 1, correcting upwards the low tdr value calculated for $Z_{i,j} = 0$.

Analysis of dense effects

When plotting the p-values for individual studies in the cancer data that we analyzed (**Supplementary Figure 3** and **Supplementary Figure 4**) many studies seem to have very high proportion of non-null realizations. In order to evaluate the performance of locfdr and normix in this situation we performed the following analysis.

We analyzed the GSE10072 study, which shows a very uneven p-value distribution (**Supplementary Text Figure A**). Here, using either normix or locfdr with estimation of the empirical null distribution resulted in very high π_0 estimates that seem to shrink the non-null group (i.e., $\pi_0 \geq 0.95$). On the other hand, when we used the theoretical null these estimations decreased substantially. As most of the density of the p-value distribution is concentrated at bins close to 0 or 1, the theoretical null estimation seems more suitable, see **Supplementary Text Figure A; C,D**. When we tried to use locfdr with theoretical null for all 29 studies of the DEG dataset, the algorithm failed to produce any output in 19 cases, even after we modified the default parameters, such as the number of degrees of freedom allowed, or the curve fitting method. For the reasons above we chose to use the normix approach with a fixed theoretical null for the analyses of the real datasets.

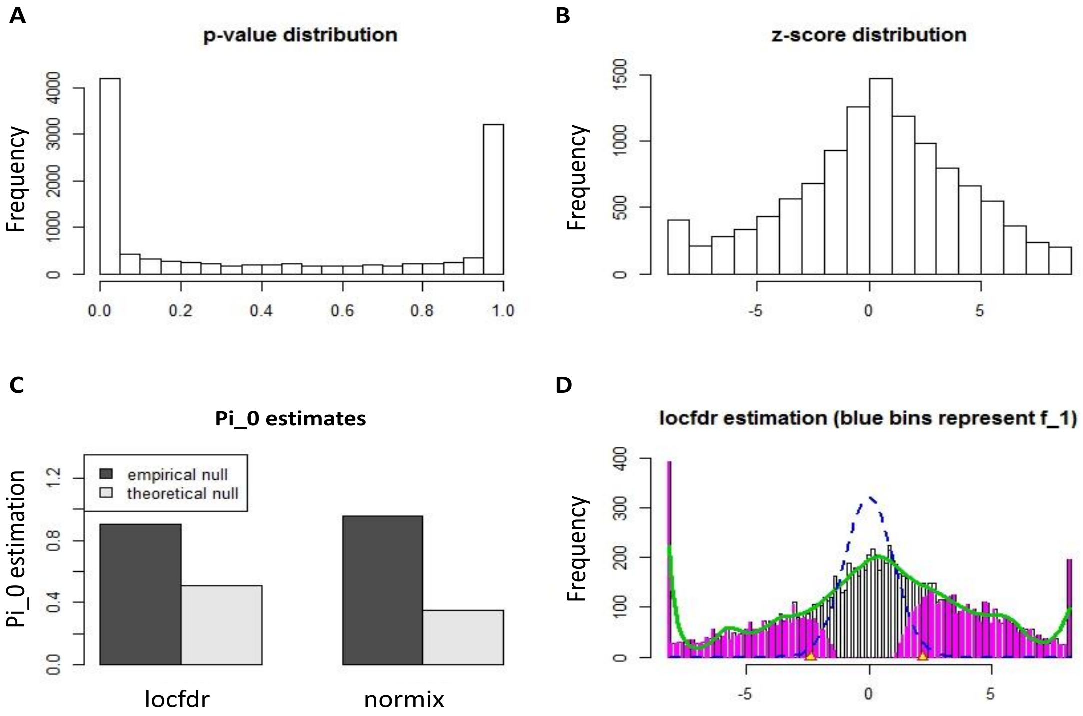

Supplementary Text Figure A Analysis of the GSE10072 dataset. A) histogram of the gene p-values. B) histogram of the gene z-scores. C) π_0 estimates using the two groups estimation algorithms with and without using the theoretical null. Empirical null estimation shrinks the non-null probability, whereas using the theoretical null captures the high percentage of non-null realizations. D) Standard plot of the locfdr estimation using the theoretical null. The plot shows the z-scores histogram with pink bins representing f_1. The dotted blue line is the standard normal distribution. The green line represents the estimated f distribution. The yellow triangles indicate threshold values with $fdr(z) \leq 0.2$.

We also performed additional simulations in order to explore the scenario of dense effects. Using the notation from the main text, we simulated $n = 5000$ genes over $m = 30$ studies. Studies 1-10 had no non-null realizations and studies 11-30 had 3000 (60% of the genes). Studies 11-20 were all independent, and studies 21-30 formed a single cluster with $r = 0.8$. To mimic the dense effects observed in some of the real datasets, the non-null realizations were obtained by sampling z-scores from a mixture of two normal distributions: $N(3, 3)$ (corresponding to very low p-values), and $N(-3, 3)$ (corresponding to very high p-values close to 1). This simulates a case in which the variance of f_1 results from both standard noise levels (similar to the null distribution) and high noise levels of the gene effects.

The results of the six tested algorithms on these data are shown in **Supplementary Text Figure B**. As in the GSE10072 dataset, locfdr with theoretical null did not report any output in many cases and it was therefore omitted. The figure shows that using normix with a fixed theoretical null was markedly better than all other approaches (e.g., Jaccard 0.78 with theoretical null vs. 0.58 with empirical null for SCREEN with k=7). As expected, as the null and non-null distributions are well separated in each study, most methods performed well and except for Fisher had high Jaccard scores and low FDP. SCREEN, Exp-count, Fisher, and SCREEN-ind all had high Jaccard scores with mild differences.

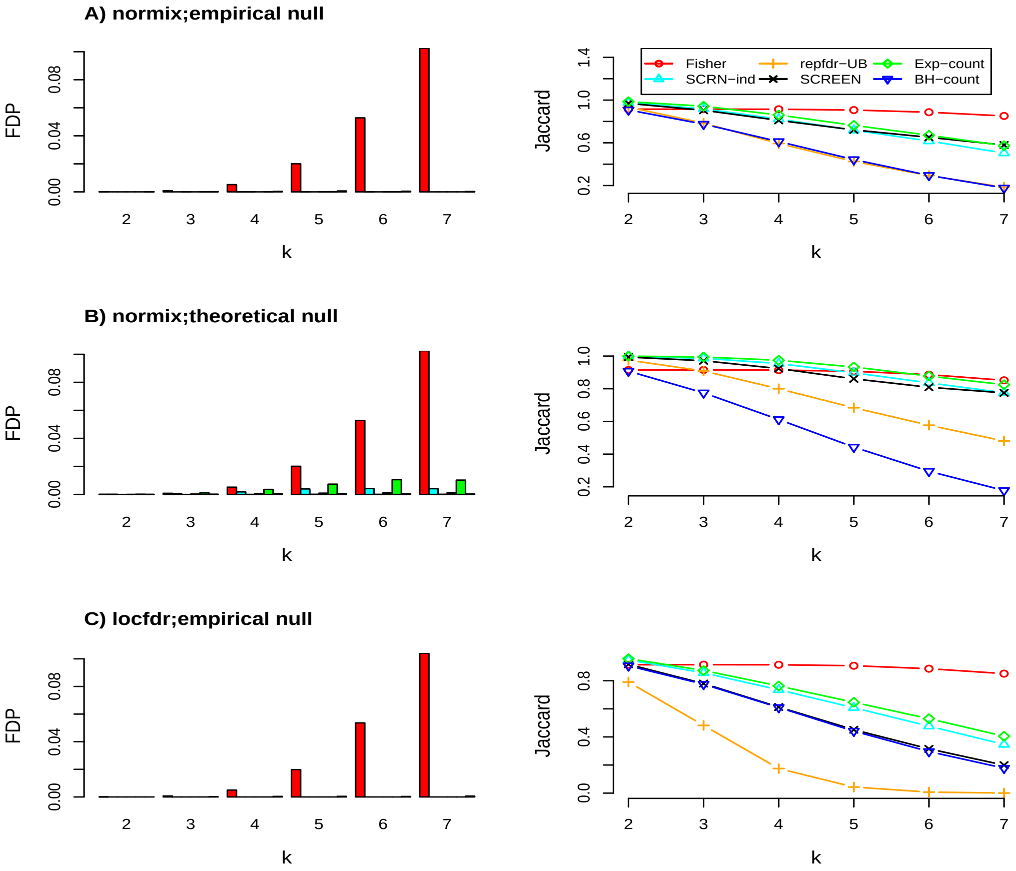

Supplementary Text Figure B Simulation results for dense effects. Simulated data on a total of 30 studies, 20 of which with dense effects; 10 form a single cluster and the rest are all independent. The left column shows the empirical FDP. The right column shows the Jaccard scores.

Extended analysis of study clusters

Here we discuss a possible extension of our clustering analysis. If the researcher is looking for recurring genes across many clusters, but with a certain coverage in each cluster, then another definition of fdr is required. More formally, we say that a gene is "interesting" in a cluster C_j if it is non-null in at least $\lceil \delta |C_j| \rceil$ of the studies, where $\delta \in (0,1]$. We say that a gene is "interesting" across clusters if it is interesting in at least d clusters. Therefore, the rejection area for this analysis is determined by the two parameters δ and d.

Let $\mathcal{H}_{\delta,d}$ be the group of configuration vectors in the null group:

$$\mathcal{H}_{\delta,d} = \{h : |\{C_j ; |h_{C_j}| < \delta |C_j|\}| < d\}$$

Then, the fdr is defined as:

$$fdr_{\delta,d}(Z_{i,\cdot}) = \sum_{h \in \mathcal{H}_{\delta,d}} \frac{P(Z_{i,\cdot}|h) \prod_{j=1}^{M} P(h_{C_j})}{P(Z_{i,\cdot})} = \sum_{h \in \mathcal{H}_{\delta,d}} \prod_{j=1}^{M} \frac{P(Z_{i,C_j}|h_{C_j}) P(h_{C_j})}{P(Z_{i,C_j})}$$

Focusing on the first cluster, partition $\mathcal{H}_{\delta,d}$ into two groups: $\mathcal{H}_1^1 = \{h : h \in \mathcal{H}_{\delta,d} \wedge |h_{C_1}| < \delta |C_1|\}|\}$, and $\mathcal{H}_1^1 = \{h : h \in \mathcal{H}_{\delta,d} \wedge |h_{C_1}| \geq \delta |C_1|\}|\}$. Thus, we get:

$$fdr_{\delta,d}(Z_{i,\cdot}) = \sum_{h \in \mathcal{H}_1^1} \frac{P(Z_{i,C_1}|h_{C_1}) P(h_{C_1})}{P(Z_{i,C_1})} fdr_{\delta,d}(Z_2, \cdots, Z_m) +$$

$$\sum_{h \in \mathcal{H}_1^2} \frac{P(Z_{i,C_1}|h_{C_1}) P(h_{C_1})}{P(Z_{i,C_1})} fdr_{\delta,d-1}(Z_2, \cdots, Z_m)$$

The formula above can be applied recursively for each cluster, which suggests a dynamic programming calculation similar to our main algorithms. As in SCREEN, this calculation requires using the EM approach within each cluster.

This formulation requires a much more complex parameterization of the null group, such as determining the suitable δ for the application at hand. We expect that such formulation can be useful in future studies that will merge a large number of study clusters of similar sizes.
