# Supplementary material for: Extracting replicable associations across multiple studies: Empirical Bayes algorithms for controlling the false discovery rate
Source: PLoS Comput Biol. 2017 Aug 18;13(8):e1005700. doi: 10.1371/journal.pcbi.1005700 (PMC5576761; doi:10.1371/journal.pcbi.1005700)
Supplement: S1 Table — (PDF) [file pcbi.1005700.s011.pdf]

Table S1 The studies of the cancer DEG dataset

GEO ID [ref]	#cancer	#control	description
GSE2549 [1]	42	11	Malignant pleural mesothelioma
GSE2719 [2]	39	15	Soft tissue sarcoma (multiple tissues)
GSE4107 [3]	10	10	Colonic mucosa from colorectal cancer patients and controls
GSE4115 [4]	79	73	Bronchial Epithelium from smokers with or without lung cancer
GSE4290 [5]	157	23	Different types of glioma vs healthy controls
GSE5764 [6]	10	20	Invasive lobular and ductal breast carcinomas
GSE6344 [7]	12	12	Kidney cancer
GSE6691 [8]	43	13	B cells from several hematopoeitic cancer types vs healthy B-cells or plasma cells
GSE7803 [9]	31	10	Cervical squamous cell carcinomas
GSE8671 [10]	32	32	Colorectal adenoma
GSE9476 [11]	26	38	Acute myeloid leukemia
GSE9574 [12]	15	14	Breast epithilium, breast cancer
GSE9750 [13]	33	33	Cervical cancer
GSE10072 [14]	58	49	Lung adenocarcinoma (smoking individuals)
GSE12452 [15]	31	10	Nasopharyngeal carcinoma
GSE12453 [16]	42	20	Lymphocyte-predominant Hodgkin lymphoma
GSE14245 [17]	12	12	Saliva, Pancreatic cancer vs controls
GSE14407 [18]	12	12	Ovarian cancer
GSE14520 [19]	22	24	Hepatocellular carcinoma
GSE19804 [20]	60	60	Non-smoking lung cancer
GSE20189 [21]	73	80	Peripheral whole blood, lung cancer
GSE20347 [22]	17	17	Esophageal squamous cell carcinoma
GSE20437 [23]	18	24	Epithelium from breast cancer patients and cancer-free prophylactic mastectomy patients
GSE22529 [24]	41	11	Chronic lymphocytic leukemia
GSE26566 [25]	104	59	Cholangiocarcinoma
GSE26910 [26]	12	12	Stroma, breast and prostate cancers vs. controls
GSE27562 [27]	57	105	PBMCs from breast cancer patients and controls
GSE28735 [28]	45	45	Pancreatic cancer
GSE32665 [29]	87	92	Lung adenocarcinoma

References

[1] D. J. Sugarbaker, R. Bueno, G. J. Gordon, G. N. Rockwell, R. V. Jensen, J. G. Rheinwald, J. N. Glickman, J. P. Aronson, B. J. Pottorf, M. D. Nitz, and W. G. Richards, "Identification of Novel Candidate Oncogenes and Tumor Suppressors in Malignant Pleural Mesothelioma Using Large-Scale Transcriptional Profiling," *Am J Pathol*, vol. 166, pp. 1827–1840, 2005.

[2] K. Y. Detwiller, N. T. Fernando, N. H. Segal, S. W. Ryeom, P. A. D'Amore, and S. S. Yoon, "Analysis of hypoxia-related gene expression in sarcomas and effect of hypoxia on RNA interference of vascular endothelial cell growth factor A," *Cancer Research*, vol. 65, pp. 5881–5889, 2005.

[3] Y. Hong, S. H. Kok, W. E. Kong, and Y. C. Peh, "A susceptibility gene set for early onset colorectal cancer that integrates diverse signaling pathways: Implication for tumorigenesis," *Clinical Cancer Research*, vol. 13, no. 4, pp. 1107–1114, 2007.

[4] A. Spira, J. E. Beane, V. Shah, K. Steiling, G. Liu, F. Schembri, S. Gilman, Y. Dumas, P. Calner, P. Sebastiani, S. Sridar, J. Beamis, C. Lamb, T. ANderson, N. Gerry, J. Keane, M. Lenburg, and J. Brody, "Airway epithelial gene expression in the diagnostic evaluation of smokers with suspect lung cancer," *Nature Medicine*, vol. 13, no. 3, pp. 361–366, 2007.

[5] L. Sun, A. M. Hui, Q. Su, A. Vortmeyer, Y. Kotliarov, S. Pastorino, A. Passaniti, J. Menon, J. Walling, R. Bailey, M. Rosenblum, T. Mikkelsen, and H. A. Fine, "Neuronal and glioma-derived stem cell factor induces angiogenesis within the brain," *Cancer Cell*, vol. 9, no. 4, pp. 287–300, 2006.

[6] G. Turashvili, J. Bouchal, K. Baumforth, W. Wei, M. Dziechciarkova, J. Ehrmann, J. Klein, E. Fridman, J. Skarda, J. Srovnal, M. Hajduch, P. Murray, and Z. Kolar, "Novel markers for differentiation of lobular and ductal invasive breast carcinomas by laser microdissection and microarray analysis.," *BMC cancer*, vol. 7, p. 55, 2007.

[7] M. L. Gumz, H. Zou, P. A. Kreinest, A. C. Childs, L. S. Belmonte, S. N. LeGrand, K. J. Wu, B. A. Luxon, M. Sinha, A. S. Parker, L. Z. Sun, D. A. Ahlquist, C. G. Wood, and J. A. Copland, "Secreted frizzled-related protein 1 loss contributes to tumor phenotype of clear cell renal cell carcinoma," *Clinical Cancer Research*, vol. 13, no. 16, pp. 4740–4749, 2007.

[8] N. C. Gutierrez, E. M. Ocio, J. de las Rivas, P. Maiso, M. Delgado, E. Ferminan, M. J. Arcos, M. L. Sanchez, J. M. Hernandez, and J. F. San Miguel, "Gene expression profiling of B lymphocytes and plasma cells from Waldenstrom's macroglobulinemia: comparison with expression patterns of the same cell counterparts from chronic lymphocytic leukemia, multiple myeloma and normal individuals," *Leukemia*, vol. 21, no. 3, pp. 541–549, 2007.

[9] Y. Zhai, R. Kuick, B. Nan, I. Ota, S. J. Weiss, C. L. Trimble, E. R. Fearon, and K. R. Cho, "Gene expression analysis of preinvasive and invasive cervical squamous cell carcinomas identifies HOXC10 as a key mediator of invasion," *Cancer Research*, vol. 67, no. 21, pp. 10163–10172, 2007.

[10] J. Sabates-Bellver, L. G. Van der Flier, M. de Palo, E. Cattaneo, C. Maake, H. Rehrauer, E. Laczko, M. a. Kurowski, J. M. Bujnicki, M. Menigatti, J. Luz, T. V. Ranalli, V. Gomes, A. Pastorelli, R. Faggiani, M. Anti, J. Jiricny, H. Clevers, and G. Marra, "Transcriptome profile of human colorectal adenomas," *Molecular cancer research : MCR*, vol. 5, no. 12, pp. 1263–1275, 2007.

[11] D. L. Stirewalt, S. Meshinchi, K. J. Kopecky, W. Fan, E. L. Pogosova-Agadjanyan, J. H. Engel, M. R. Cronk, K. S. Dorcy, A. R. McQuary, D. Hockenbery, B. Wood, S. Heimfeld, and J. P. Radich, "Identification of genes with abnormal expression changes in acute myeloid leukemia," *Genes, Chromosomes {&} Cancer*, vol. 47, no. 1, pp. 8–20, 2008.

[12] A. Tripathi, C. King, A. De La Morenas, V. K. Perry, B. Burke, G. A. Antoine, E. F. Hirsch, M. Kavanah, J. Mendez, M. Stone, N. P. Gerry, M. E. Lenburg, and C. L. Rosenberg, "Gene expression abnormalities in histologically normal breast epithelium of breast cancer patients," *International Journal of Cancer*, vol. 122, no. 7, pp. 1557–1566, 2008.

[13] L. Scotto, G. Narayan, S. V. Nandula, H. Arias-Pulido, S. Subramaniyam, A. Schneider, A. M. Kaufmann, J. D. Wright, B. Pothuri, M. Mansukhani, and V. V. Murty, "Identification of copy number gain and overexpressed genes on chromosome arm 20q by an integrative genomic approach in cervical cancer: Potential role in progression," *Genes Chromosomes and Cancer*, vol. 47, no. 9, pp. 755–765, 2008.

[14] M. T. Landi, T. Dracheva, M. Rotunno, J. D. Figueroa, H. Liu, A. Dasgupta, F. E. Mann, J. Fukuoka, M. Hames, A. W. Bergen, S. E. Murphy, P. Yang, A. C. Pesatori, D. Consonni, P. A. Bertazzi, S. Wacholder, J. H. Shih, N. E. Caporaso, and J. Jen, "Gene expression signature of cigarette smoking and its role in lung adenocarcinoma development and survival," *PLoS ONE*, vol. 3, 2008.

[15] S. Sengupta, J. a. den Boon, I.-H. Chen, M. a. Newton, D. B. Dahl, M. Chen, Y.-J. Cheng, W. H. Westra, C.-J. Chen, A. Hildesheim, B. Sugden, and P. Ahlquist, "Genome-wide expression profiling reveals EBV-associated inhibition of MHC class I expression in nasopharyngeal carcinoma.," *Cancer research*, vol. 66, no. 16, pp. 7999–8006, 2006.

[16] V. Brune, E. Tiacci, I. Pfeil, C. Döring, S. Eckerle, C. J. M. van Noesel, W. Klapper, B. Falini, A. von Heydebreck, D. Metzler, A. Bräuninger, M.-L. Hansmann, and R. Küppers, "Origin and pathogenesis of nodular lymphocyte-predominant Hodgkin lymphoma as revealed by global gene expression analysis.," *The Journal of experimental medicine*, vol. 205, no. 10, pp. 2251–2268, 2008.

[17] L. Zhang, J. J. Farrell, H. Zhou, D. Elashoff, D. Akin, N. H. Park, D. Chia, and D. T. Wong, "Salivary Transcriptomic Biomarkers for Detection of Resectable Pancreatic Cancer," *Gastroenterology*, vol. 138, no. 3, 2010.

[18] N. J. Bowen, L. D. Walker, L. V. Matyunina, S. Logani, K. a. Totten, B. B. Benigno, and J. F. McDonald, "Gene expression profiling supports the hypothesis that human ovarian surface epithelia are multipotent and capable of serving as ovarian cancer initiating cells.," *BMC medical genomics*, vol. 2, p. 71, 2009.

[19] S. Roessler, H. L. Jia, A. Budhu, M. Forgues, Q. H. Ye, J. S. Lee, S. S. Thorgeirsson, Z. Sun, Z. Y. Tang, L. X. Qin, and X. W. Wang, "A unique metastasis gene signature enables prediction of tumor relapse in early-stage hepatocellular carcinoma patients," *Cancer Research*, vol. 70, no. 24, pp. 10202–10212, 2010.

[20] T. P. Lu, M. H. Tsai, J. M. Lee, C. P. Hsu, P. C. Chen, C. W. Lin, J. Y. Shih, P. C. Yang, C. K. Hsiao, L. C. Lai, and E. Y. Chuang, "Identification of a novel biomarker, SEMA5A, for non-small cell lung carcinoma in nonsmoking women," *Cancer Epidemiol Biomarkers Prev*, vol. 19, no. 10, pp. 2590–2597, 2010.

[21] M. Rotunno, N. Hu, H. Su, C. Wang, A. M. Goldstein, A. W. Bergen, D. Consonni, A. C. Pesatori, P. A. Bertazzi, S. Wacholder, J. Shih, N. E. Caporaso, P. R. Taylor, and M. T. Landi, "A gene expression signature from peripheral whole blood for stage I lung adenocarcinoma.," *Cancer prevention research (Philadelphia, Pa.)*, vol. 4, no. 10, pp. 1599–1608, 2011.

[22] N. Hu, R. J. Clifford, H. H. Yang, C. Wang, A. M. Goldstein, T. Ding, P. R. Taylor, and M. P. Lee, "Genome wide analysis of DNA copy number neutral loss of heterozygosity (CNNLOH) and its relation to gene expression in esophageal squamous cell carcinoma.," *BMC genomics*, vol. 11, p. 576, 2010.

[23] K. Graham, A. de las Morenas, A. Tripathi, C. King, M. Kavanah, J. Mendez, M. Stone, J. Slama, M. Miller, G. Antoine, H. Willers, P. Sebastiani, and C. L. Rosenberg, "Gene expression in histologically normal epithelium from breast cancer patients and from cancer-free prophylactic mastectomy patients shares a similar profile.," *British journal of cancer*, vol. 102, no. 8, pp. 1284–1293, 2010.

[24] A. Gutierrez, R. C. Tschumper, X. Wu, T. D. Shanafelt, J. Eckel-Passow, P. M. Huddleston, S. L. Slager, N. E. Kay, and D. F. Jelinek, "LEF-1 is a prosurvival factor in chronic lymphocytic leukemia and is expressed in the preleukemic state of monoclonal B-cell lymphocytosis," *Blood*, vol. 116, no. 16, pp. 2975–2983, 2010.

[25] J. B. Andersen, B. Spee, B. R. Blechacz, I. Avital, M. Komuta, A. Barbour, E. A. Conner, M. C. Gillen, T. Roskams, L. R. Roberts, V. M. Factor, and S. S. Thorgeirsson, "Genomic and genetic characterization of cholangiocarcinoma identifies therapeutic targets for tyrosine kinase inhibitors," *Gastroenterology*, vol. 142, no. 4, 2012.

[26] A. Planche, M. Bacac, P. Provero, C. Fusco, M. Delorenzi, J. C. Stehle, and I. Stamenkovic, "Identification of prognostic molecular features in the reactive stroma of human breast and prostate cancer," *PLoS ONE*, vol. 6, no. 5, 2011.

[27] H. G. LaBreche, J. R. Nevins, and E. Huang, "Integrating Factor Analysis and a Transgenic Mouse Model to Reveal a Peripheral Blood Predictor of Breast Tumors," *BMC Medical Genomics*, vol. 4, no. 1, pp. 1–14, 2011.

[28] G. Zhang, P. He, H. Tan, A. Budhu, J. Gaedcke, B. Michael Ghadimi, T. Ried, H. G. Yfantis, D. H. Lee, A. Maitra, N. Hanna, H. Richard Alexander, and S. Perwez Hussain, "Integration of metabolomics and transcriptomics revealed a fatty acid network exerting growth inhibitory effects in human pancreatic cancer," *Clinical Cancer Research*, vol. 19, no. 18, pp. 4983–4993, 2013.

[29] I.-J. J. Kim, D. Quigley, M. D. To, P. Pham, K. Lin, B. Jo, K.-Y. Y. Jen, D. Raz, J. Kim, J.-H. H. Mao, D. Jablons, and A. Balmain, "Rewiring of human lung cell lineage and mitotic networks in lung adenocarcinomas," *Nat Commun*, vol. 4, p. 1701, 2013.
